# Blue or gold? Visual perception and the restoration of a Medieval painting

**DOI:** 10.1177/03010066261433859

**Published:** 2026-03-29

**Authors:** Feline A. van Gelderen, Yuexi Zhu, Ignace T.C. Hooge, Sanne Frequin, Roy S. Hessels

**Affiliations:** 1 Department of Experimental Psychology, Helmholtz Institute, Utrecht University, Utrecht, the Netherlands; 2 Department of History and Art History, Utrecht University, Utrecht, the Netherlands

**Keywords:** gaze, art, gist perception, art conservation, eye tracking

## Abstract

This study investigates how the background color of the *Lindau Crucifixion*, a fifteenth-century panel painting by the Master of the Lamentation of Christ in Lindau, affects visual perception. Originally gilded, the painting was later overpainted with a blue paint, raising questions in conservation about whether to preserve the intervention or restore the gold. To assess potential perceptual consequences, we used digital reconstructions of the painting in eye-tracking and gist perception experiments. In the eye-tracking task, participants viewed each version for 6 s among similar medieval paintings. Faces were consistently gazed at, while angels attracted less gaze. However, a slightly reduced likelihood of fixating angels was found for the blue background. In the gist perception task, participants described similar elements across both versions, reporting the central figures regardless of background color. These findings suggest limited perceptual differences under brief viewing, highlighting the potential of perceptual experiments to inform art conservation decisions.

## Introduction

**Figure 1.**
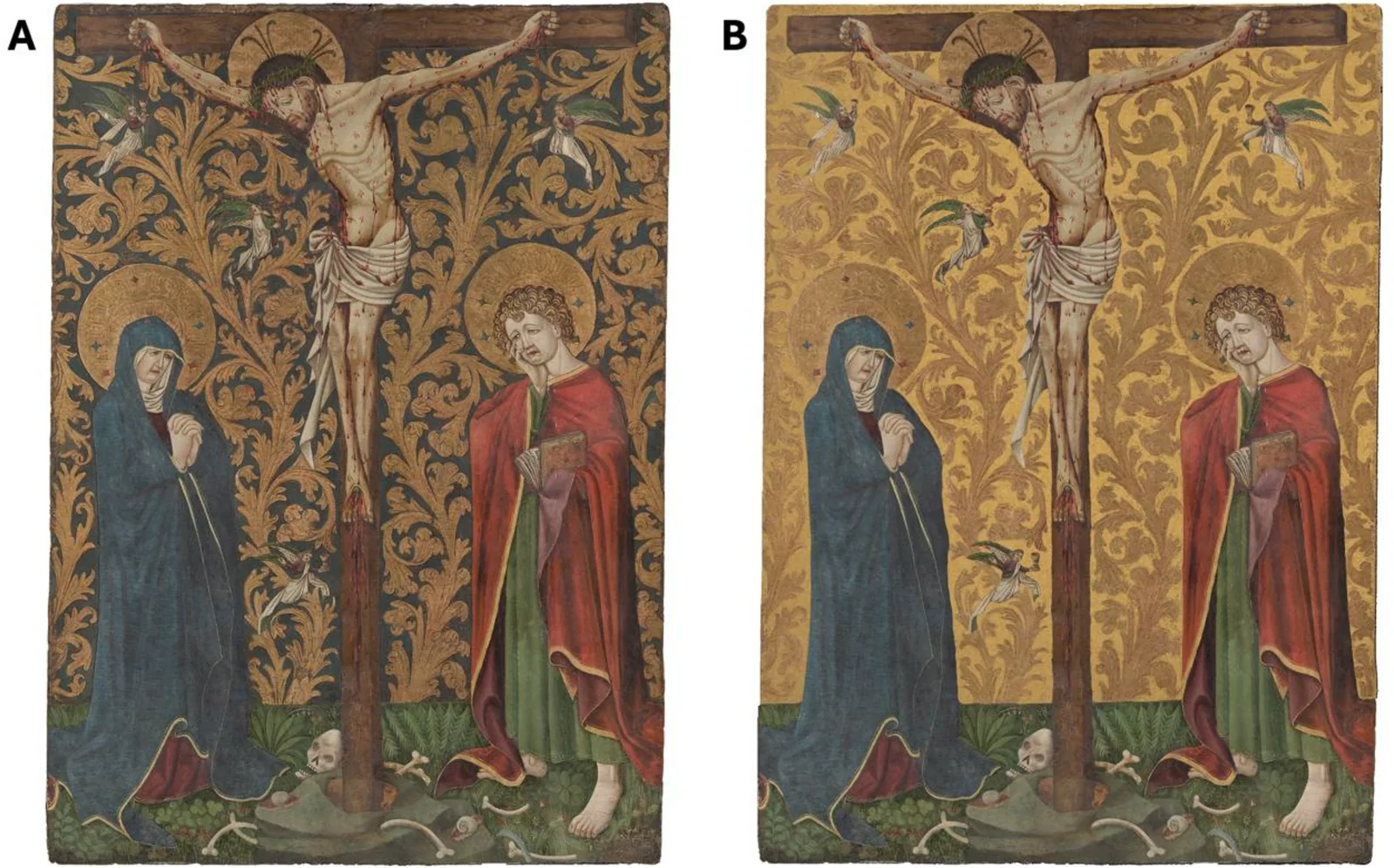
Photograph of the Lindau Crucifixion (ca. 1425), Museum Catharijneconvent Utrecht, photo Factum Foundation (panel A). Digital reconstruction representing the fully gilded background, ^©^ Ruben Wiersma (panel B).

The Crucifixion of the Master of the Lamentation of Christ in Lindau (henceforth: Lindau Crucifixion, see Figure 1A) is an early fifteenth-century panel painting, part of the collection of Museum Catharijneconvent in Utrecht, the Netherlands. The painting shows the crucified Christ, flanked by his mother Mary and Saint John the Evangelist. Four small angels catch Christ's blood in golden chalices, a significant iconographic detail representing the transubstantiation. At the bottom of the cross lay bones and a skull, referencing the site Christ was crucified: Golgotha (Matthew 27:33, Mark 15:22, Luke 23:33, and John 19:17). The figures are set against a blue ground adorned with golden tendrils.

When the painting was brought out of the depot for restoration in 2020, the conservator discovered that the blue ground was an overpaint which obscures a once fully gilded surface (Figure 1B). This discovery posed a dilemma: should this later intervention be preserved as part of the artwork's historical life, or should the original gold be restored to approximate the artist's intent? In conservation ethics, it is emphasized that the conservator's perspective is not the only one that matters. The restoration of an object must also consider the interests of future generations who will engage with and appreciate the artwork. Consequently, conservation decisions should be made with these future users in mind. Restoration decisions should therefore be reversible, and the interventions should be minimal (Muñoz Viñas, 2005). The removal of the blue background would constitute a large and irreversible intervention, thereby depriving future audiences of access to this layer of meaning.

However, this deprives the audience of an important aspect of the painting: the golden background. In the medieval period, gold conveyed opulence, authority, and divine symbolism through various artistic mediums like altar pieces, sculptures, and manuscripts. Beyond its remarkable malleability, gold assumed a crucial role in heightening the mystique surrounding art works (Kim, 2019). On the painting, this effect is now diminished by the later application of blue overpaint. To potentially help resolve this dilemma, two digital reconstructions (see Figure 1) and two high-quality facsimiles were created (Tissen et al., 2023), which presents a unique opportunity for comparison.

In this study, we used the digital reconstructions of the Lindau Crucifixion to investigate two potential consequences of the background color: (a) how it affects viewers’ gaze allocation, and (b) how it affects what viewers perceive from the painting at a glance. Gaze allocation was investigated using eye tracking during a memory and recognition test. Perception at a glance was investigated using an online gist perception experiment. Regarding gaze allocation, we may expect that regardless of the background color, faces capture and maintain attention and gaze disproportionally (e.g., Bindemann et al., 2005; Birmingham et al., 2008a, 2008b; Langton et al., 2008; Theeuwes & van der Stigchel, 2006). At the same time, we may expect that the blue background color creates more clutter in the background and weaker contrasts with the angels, thereby reducing their relative attention-attraction power (e.g., Itti & Koch, 2000). Regarding perception at a glance, humans are remarkably capable of extracting scene gist under very brief presentations (Oliva, 2005; Thorpe et al., 1996) and report substantial detail in scenes for presentation durations around 0.5 s (e.g., Fei-Fei et al., 2007; Hessels et al., 2021). It has further been reported that observers start reporting holistic aspects of famous paintings within 2 s of stimulus presentation (Locher et al., 2007). Thus, we expect observers to report substantial detail after the presentation of a few seconds. We investigate whether certain aspects are mentioned more often for the blue or gold background, for example, the angels.

The goal of the present study is twofold. First, it demonstrates the potential consequences of the choice of background color in the restoration process for gaze allocation and perception at a glance of the Lindau Crucifixion. Second, more generally, it attests to the utility of common experiments in perception research for the process of art restoration, which others may wish to exploit in the future.

## Method

### Experiment 1—Gaze Allocation

#### Participants

Participants were recruited among the Psychology students at Utrecht University, and the authors’ network. When eligible, psychology students were compensated with course credit (participant hours). Forty-two participants completed the eye-tracking experiment. Two participants were excluded, because the inaccuracy of the eye-tracking data exceeded 2° (n = 1) or because they failed to comply with the instructions of the experiment (n = 1). The sample size was based (a) on empirical guidelines for scene-viewing studies, describing progressively diminishing returns after 20–30 participants (Hoogerbrugge et al., 2025) and (b) on a similar previous study who included 37 participants per group in a between-participant study (Leonards et al., 2007). The mean age of the included participants was 23.5 years (range 19–29 years). All participants reported normal or corrected-to-normal vision. The experiment was approved by the Ethics Committee of the Faculty of Social and Behavioral Sciences at Utrecht University (Protocol 25-1822). Participants gave informed consent prior to starting the experiment.

#### Stimuli, Apparatus, and Procedure

As task instructions may impact the allocation of gaze, and a free-viewing experiment may result in participants interpreting the instructions in various ways (see, e.g., Hooge et al., 2025, p. 16), we hid the two versions of the painting in a recognition experiment consisting of 16 memory trials, and 16 recognition trials. For the 16 memory trials, both versions of the painting (blue background and gold background) were presented in addition to seven pairs of similarly matching paintings. These were added as fillers (see full details of fillers and sources in the Supplementary Material) to avoid participants’ awareness of the manipulation of the background color and the goal of the study.

The digital reconstructions of the Lindau Crucifixion presented in Figure 1 were used to guarantee that all visual features of the two images other than the adjusted background color were identical. Filler paintings were selected to be visually similar according to the following criteria: (a) dated from the 14th or 15th century, (b) painted using the tempera technique, and (c) works with Christian iconography. Matching pairs were curated by selecting pieces from the same panel or series, or pieces depicting the same biblical subject(s). The purpose of including these filler paintings was to prevent participants from focusing exclusively on the Lindau Crucifixion pair. All stimuli were displayed at full vertical height and centered on a gray background (1,920 × 1,080 pixels). For the Lindau Crucifixion this translated to approximately 31°×22° of the visual field, corresponding to a 2.3 m real-world viewing distance of the painting.

All pairs were split up so that one painting of each pair was presented in the first eight, and in one in the last eight trials. We opted for a within-subjects design instead of a between-subjects design to reduce the influence of individual variation in terms of, for example, face bias, on the between-version comparison. The first appearance of the Lindau Crucifixion was deliberately not at the start of the experiment, so that participants were well-accustomed once it appeared. The background color of the first presentation (gold or blue) was counterbalanced across participants. The order of the filler pairs was determined at random once and fixed for all participants.

Participants were instructed to look at the artworks carefully and remember them in preparation for a subsequent recognition test. A central fixation cross appeared before every painting, which the participant confirmed to be looking at by pressing the spacebar. Then the painting was shown for 6 s. After the 16 images had been viewed, a recognition test of 16 trials was conducted, with eight previously shown images (one selected from each pair at random) and eight new filler paintings.

The eye tracking experiment took place in a brightly lit room with black walls, generally used for psychophysics experiments. After general instruction and providing informed consent, participants sat in an office chair of adjustable height, with their heads placed in a chin and forehead rest for the 16 memory trials of the recognition experiment. A Tobii Pro Nano was used to track gaze location on the screen at 60 Hz for all memory trials in the recognition experiment. Viewing distance was approximately 62 cm, while the distance between eye tracker and participants’ eyes was approximately 65 cm. A 27-inch Asus PG278Q monitor was used for stimulus presentation at a resolution of 1,920 by 1,080 pixels and 60 Hz refresh rate. After positioning the participant in front of the eye tracker and screen, an automatic 9-point calibration procedure was carried out. The cut-off for successful calibration and initiation of the experimental task was set at an accuracy of 1°. If this was not reached, the calibration procedure was repeated. The 16 memory trials of the experiment were presented with the Tobii Pro Lab software (Version 24.21). After completing the 16 memory trials, the participants were moved to an adjacent desk where the 16 recognition trials of the experiment were presented using Gorilla (Anwyl-Irvine et al., 2020) on a separate laptop.

At the end of the experiment, participants were presented with a brief questionnaire to assess what they thought the focus of the study was (open question). Additionally, participants rated their knowledge of art history and Christian symbolism. This was done on a 5-point Likert scale (1 = *not at all*, 5 = *very much*) in response to the following questions: (1) To what extent are you knowledgeable about art history in general? (2) To what extent are you familiar with Christian symbolism (e.g., the meaning of symbols such as the cross and the lamb)? (3) To what extent are you familiar with stories from the Bible?

#### Data Analysis

**Figure 2.**
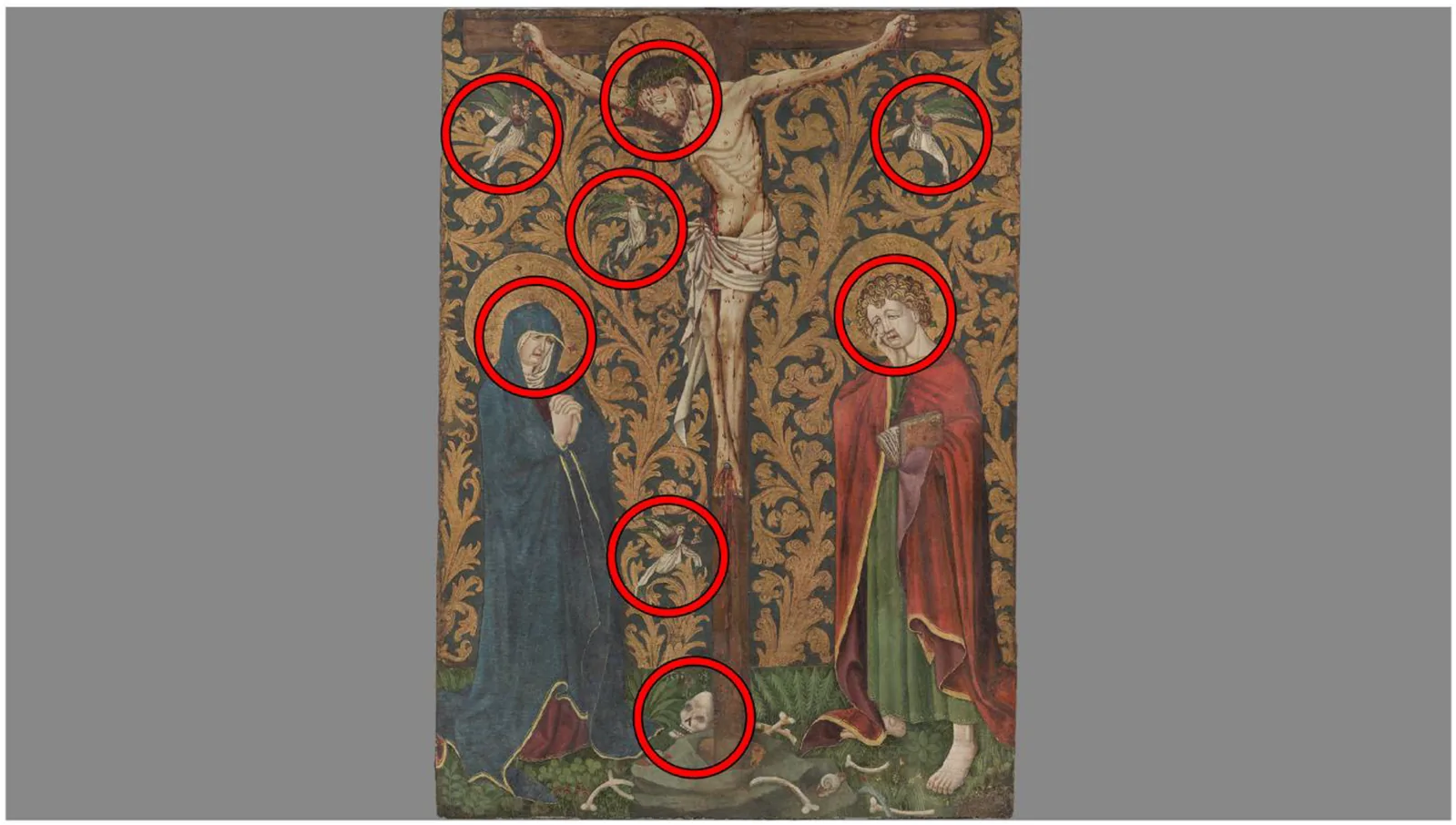
Areas of interest (AOIs) defined for both versions of the Lindau Crucifixion. The AOIs correspond to the four faces and four angels depicted in the painting.

We only consider eye-tracking data for the two versions of the Lindau Crucifixion. The following steps were taken to transform the eye tracking data into interpretable outcomes. First, fixations were classified using the identification by two-means clustering (I2MC) algorithm v2.0 (Hessels et al., 2017). Areas Of Interest (AOI) were defined for eight objects: the four angels and the four faces in the painting (including the skull at the bottom, which we consider a face too). AOIs were operationalized as circular AOIs around handpicked centers (akin to the Limited-Radius Voronoi Tessellation method described in Hessels et al., 2016). The radius was set to 75 pixels (approximately 2.2°) and the AOIs are depicted in Figure 2. Upon experiment completion, heat maps were produced to confirm that this AOI set captures most of the gaze on the image. Each fixation was assigned to one of the eight angel or face AOIs, or to the none AOI (i.e., all space outside the other AOIs). From this, four gaze measures were computed: (a) total looking time for all faces, (b) total looking time for all angels, (c) time to first look at any face, and (d) time to first look at any angel.

Performance on the recognition test was quantified by the proportion of correct answers. Bayesian paired sample t-tests were conducted with JASP (JASP Team, 2024) to quantify the likelihood of the alternative hypothesis (blue ≠ gold) against the null hypothesis (blue = gold) given the collected data. We use the notations for Bayes Factors as given by JASP, which represent the evidence in favor of a statistical hypothesis given the data (the higher the value, the stronger the evidence). For detailed interpretation of the values, we refer the reader to Table 1 in Schönbrodt and Wagenmakers (2018).

### Experiment 2—Perception at a Glance

#### Participants

Participants were recruited as in Experiment 1. Forty total participants were recruited, with participant age ranging from 22 to 62 years. Most participants reported normal or corrected-to-normal vision, with two participants indicating minor color vision deficiencies. The experiment was approved by the Ethics Committee of the Faculty of Social and Behavioral Sciences at Utrecht University (Protocol 25-1820). Participants gave informed consent prior to starting the experiment.

#### Stimuli, Apparatus, and Procedure

The stimuli used in Experiment 2 were identical to those in Experiment 1. To probe perception at a glance, stimuli were briefly (2.5 s) presented, after which participants were asked to describe what they saw. The experiment was conducted online using Gorilla. Participants accessed the study via a secure web link and completed the task using their own laptop or desktop computers. To ensure consistency in visual presentation and reduce environmental variability, participants were instructed to follow these standardized conditions before beginning the experiment: (a) sitting in a quiet, undisturbed space, (b) maintaining a viewing distance from the screen of approximately 50–70 cm, (c) setting their browser to 100% zoom or entering full-screen mode, and (d) adjusting their display brightness to the maximum level.

Upon opening the experiment link, participants were first presented with an information letter and consent form. Hereafter, participants completed a brief screening survey, indicating their age and whether they wore glasses or contact lenses. They were also asked to follow the viewing instructions described above. The experiment then began with an instruction screen explaining the task: participants would view each artwork briefly and then describe in detail what they had seen each time. Before each image, a central white fixation dot was presented. Participants were instructed to press the spacebar while fixating on the dot. After a 0.5 s inter-stimulus interval during which the fixation dot was black instead of white, the painting was displayed. Each painting was displayed on screen for 2.5 s. Hereafter, participants were asked to type a description of what they had seen in a response box. This structure was repeated across all 16 images. The order of stimuli was identical to experiment 1, with (a) the first Lindau Crucifixion not placed at the start, (b) the first Lindau Crucifixion presentation (gold or blue) counterbalanced across participants, and (c) the order of the filler pairs fixed.

Finally, the same questionnaire on the focus of the study and the participants familiarity with art history and Christian symbolism as in Experiment 1 was presented.

#### Data Analysis

A coding scheme was developed to annotate participants’ written descriptions of the two versions of the Lindau Crucifixion painting. The aim of the coding process was to filter the diverse textual responses into a coherent set of thematic categories that could capture recurring interpretive elements across the dataset. Codes were refined through repeated rounds of trial coding and discussion between the authors. The final scheme included 13 binary-coded categories, grouped the figures (e.g., presence, number, behavior, detail, emotion), angels (e.g., presence, number, position, detail), other symbolic recognition such as skull and bones, and background description. An additional category marked whether participants reported having seen the painting before, relevant only for the second repetition of the Lindau Crucifixion. Multiple categories could be assigned to a single response.

To assess whether participants were more likely to comment on specific aspects of the painting depending on the background color, a series of Bayesian contingency table analyses were conducted for each coded category using JASP.

## Results

### Experiment 1—Gaze Allocation

#### Eye-Tracking Data Quality

Eye-tracking data quality was operationalized by the accuracy, precision and data loss of the gaze position signal. Accuracy ranged from 0.28° to 1.00° (*M* = 0.60°, *SD* = 0.21°), which was well below the AOI diameter (4.4°). Thus, assigning fixations to AOIs was deemed reliable.

Precision was computed per participant as the median of the root mean square sample-to-sample deviation reported by the I2MC algorithm per fixation. Across participants, this resulted in a mean precision of 0.11° (*SD* = 0.03°, range 0.07°–0.19°) for the Blue Lindau Crucifixion and 0.10° (*SD* = 0.02°, range 0.06°–0.19°) for the Gold Lindau Crucifixion. Data loss was expressed as a proportion of the trial without valid gaze coordinates. This was 0.02 (*SD* = 0.03, range 0–0.14) for the Blue Lindau Crucifixion and 0.01 (*SD* = 0.01, range 0–0.06) for the Gold Lindau Crucifixion. Precision and data loss were not deemed problematic for fixation classification, nor did we observe differences between the two versions of the Lindau Crucifixion that might impact our interpretation of any differences in gaze measures.

#### Recognition Performance

The recognition test served to check that participants complied with the instructions to inspect the artworks carefully and remember them. Inspection of participant recognition test performance revealed an outlier with performance at chance level, who was subsequently excluded on the premise that they had not complied with task instructions sufficiently. For the 40 included participants, the mean proportion of correct responses was 0.88 (*SD* = 0.10, range = 0.75–1.00).

#### Gaze Measures

**Figure 3.**
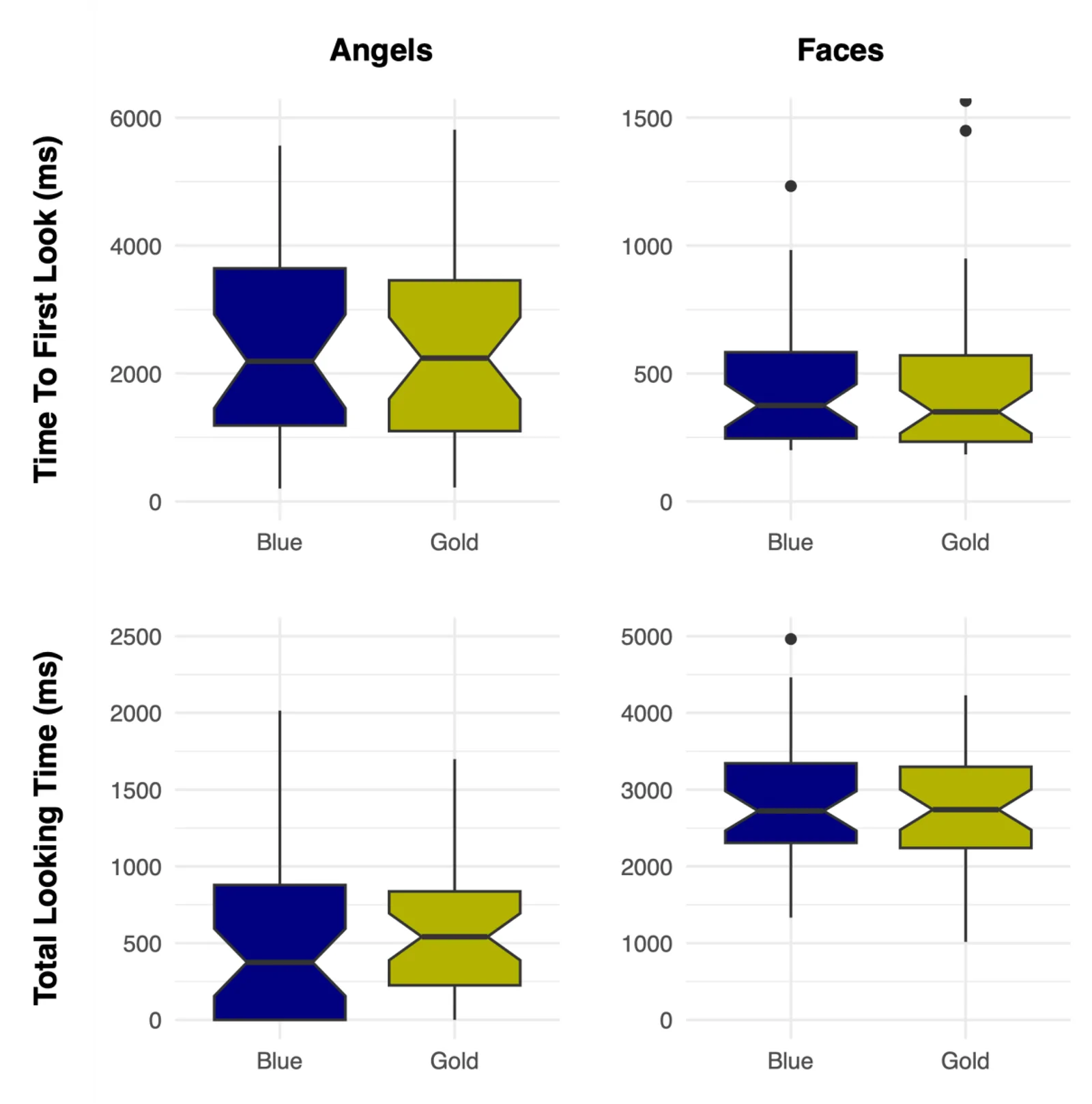
Gaze behavior for the two versions of the Lindau Crucifixion. Box and whisker plots for time to first look at any angel (top left panel) or face (top right panel) and total looking time for all angels (bottom left panel) and all faces (bottom right panel) for the two versions of the Lindau Crucifixion. Thick middle line in each box corresponds to the group median, surrounding notches give a rough 95% confidence interval.

Gaze behavior was operationalized using the time to first look at any angel or face, and the total looking time at faces and angels. Figure 3 represents the time to first look at any angel (top left panel) and any face (top right panel), as well as the total looking time to all angels (bottom left panel) and all faces (bottom right panel). Total looking time to the faces (bottom right panel) was similar across the two versions of the Lindau Crucifixion (mean duration for Gold trials 2,708 ms, *SD* = 724 ms and for Blue trials 2,786 ms, *SD* = 806 ms) and amounted to over 50% of the total fixation time within a trial (5,336 ms on average). Total looking time to the angels (bottom left panel) was slightly longer for the Gold version of the Lindau Crucifixion (mean duration for Gold trials 575 ms, *SD* = 432 ms and for Blue trials 500 ms, *SD* = 528 ms). Finally, total looking time not attributed to any of the faces or angels was comparable between conditions (mean duration for Gold trials 2,055 ms, *SD* = 890 ms and for Blue trials 2,046 ms, *SD* = 787 ms).

Note that, especially for the faces, not only total looking time but also time to first look were similar across the two versions of the Lindau Crucifixion (Figure 3, right panels). Indeed, the Bayes Factor for the null hypothesis of no difference was BF_01_ = 4.52 for the time to first look at any angel, BF_01_ = 4.16 for the total looking time at all angels. The Bayes Factor was BF_01_ = 3.74 for the time to first look at any face and BF_01_ = 5.12 for the total looking time at all faces. Thus, all four t-tests indicated moderate support for the null hypothesis that there is no difference between gaze behavior during presentation of the two versions of the Lindau Crucifixion.

**Figure 4.**
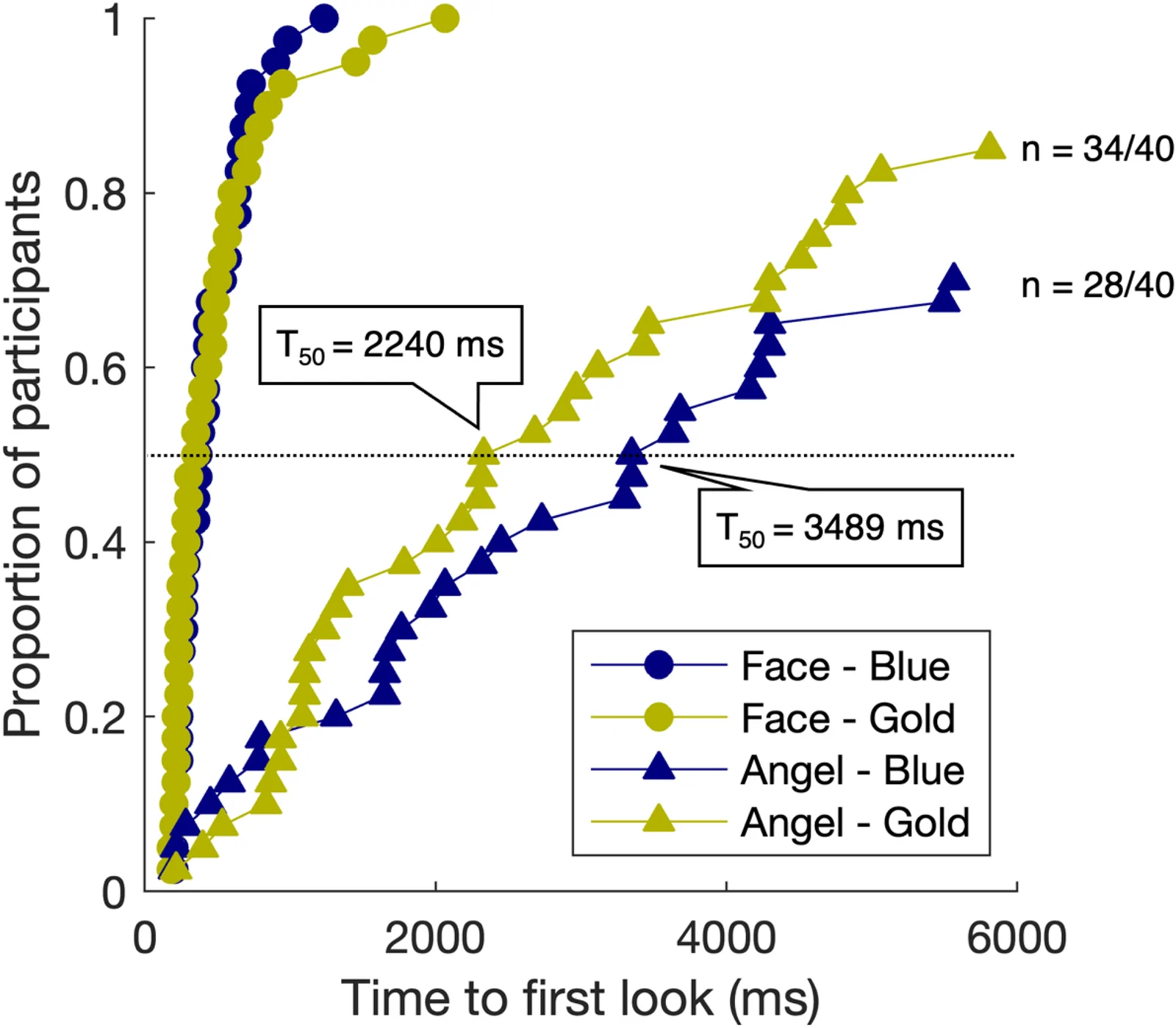
Probability of fixating any angel and the time to first look at any angel across the two versions of the Lindau Crucifixion. All participants fixated at least one face in the Blue and Gold version of the Lindau Crucifixion and distributions of time to first look overlap almost completely. Fixating any angel was less likely for the Blue than for the Gold version (participants numbers depicted on the right side of the figure). Callouts depict the bootstrapped time at which 50% of participants fixated the angel against each background (T_50_).

However, if someone did not fixate any angel or face in either the gold or blue version, a time to first look cannot be computed for that trial and they subsequently do not end up in the statistical analysis. That this may be relevant is evident from the fact that one or more fixations on any angel were recorded for 34 out of 40 participants in the gold condition, yet only 28 out of 40 participants in the blue condition. Three participants did not make any fixation on any angel across both versions, while 15 out of 40 participants made no fixations on an angel in either one of the two versions. For comparison, at least one fixation was registered on any face for all participants across both versions. One way to visualize both the probability of fixating the AOI and the time to first look at that AOI is a cumulative distribution as depicted in Figure 4. As is visible from this graph, faces were fixated by all participants for both versions of the Lindau Crucifixion, and the distribution of the time to first look at any face was virtually identical across the two versions. Angels, on the other hand, were fixated slightly less for the Blue version than for the Gold version. The time at which 50% of the participants fixated any angel (T_50,_ see Hooge & Camps, 2013) seemed slightly longer for the Blue version (bootstrapped 50th percentile = 3,489 ms, 95%CI = 2,065–4,297 ms) than for the Gold version (bootstrapped 50th percentile = 2,240 ms, 95%CI = 1,232–2,965 ms). We interpret this as evidence for minor differences in gaze allocation between the Blue and Gold version of the Lindau Crucifixion, suggesting that the angels attract gaze slightly less in the Blue version than in the Gold version.

Note that conducting the analyses with only the first iteration of each painting, that is, implementing a between-participant approach instead of a within-participant approach did not lead to altered conclusions.

### Experiment 2—Perception at a Glance

#### Inter Rater Agreement

Responses were coded by authors YZ (87.5%, all English responses) and FvG (12.5%, all Dutch responses). The latter annotated an additional 37.5% of the responses (in English) to assess inter-rater reliability. Across all categories coded by both authors, there were only two discrepancies for 30 responses (each coded using 13 categories), resulting in a percent agreement of 99.5%. Percent agreement is a straightforward reliability measure, particularly suitable when categories are clearly defined and there are only two coders (Hayes & Krippendorff, 2007).

#### Coded Categories

Table 1 depicts the frequency of each code for the Blue and Gold versions of the Lindau Crucifixion. Bayesian contingency table analyses suggest no difference in what was verbalized across the two versions of the painting. For instance, in the category “Figure” (e.g., Christ, Mary, John the Evangelist), the Bayes factor (BF_01_ = 10.38) indicates that participants were equally likely to mention the figures regardless of the version they viewed. The same pattern was observed for other categories, including the number and positions of figures, the angels, and symbolic elements. This suggests that viewers perceived and reported these elements at similar frequencies across the two conditions and extracted the same gist from the painting across the two versions. Note that it did not seem more likely for the background color or repetition to be noticed for the Blue or Gold version.

**Table 1.** Analysis of textual responses.

Coding Category	Counts Blue Version	Counts Gold Version	BF_01_
Figure (e.g., Christ, Mary, John the Evangelist)	39	40	10.38
Number of Figure	37	36	5.90
Position of Figure (e.g., on the cross, on the side)	34	34	5.08
Behavior of Figure (e.g., crying, praying, bleeding)	24	20	2.48
Detail of Figure (e.g., red/blue garment, halo)	14	10	2.52
Emotion of Figure (e.g., sad, terror)	11	9	3.71
Angels	6	6	5.08
Number of Angels	4	2	4.92
Position of Angels	5	4	5.38
Details	1	2	7.88
Other Symbolic Elements (e.g., skull, bones)	9	10	4.15
Background (e.g., color, decorative elements)	5	9	2.48
Seen this painting before	9	12	3.14

*Note*. Counts for Each Annotated Category and Outcome Bayesian Contingency Table Analyses. BF_01_ = Bayes Factor for the null hypothesis of no difference between Lindau Crucifixion versions.

#### Experiments 1 and 2—Post Task Survey

In general, participants thought that the study was designed to assess what they would recall when religious artworks were shown repeatedly. However, none of the participants explicitly identified the Lindau Crucifixion or the manipulated background (Blue vs. Gold) as the study's focus. The self-rating scores on knowledge of art history, Christian symbolism, and biblical stories were very similar across the two samples and suggested that both samples mainly consisted of participants with low to moderate familiarity with these topics. Knowledge-ratings for the eye tracking and perception experiment were respectively: 2.3 (*SD* = 1.0) versus 2.1 (*SD* = 0.9) on art history, 2.8 (*SD* = 1.1) versus 2.8 (*SD* = 1.0) on Christian symbolism, and 2.7 (*SD* = 1.1) versus 2.7 (*SD* = 1.1) on biblical stories.

## Discussion

We investigated two potential consequences of the blue or gold background color of the Lindau Crucifixion, namely how it affects viewers’ gaze allocation and what viewers perceive from the painting at a glance, using digital reconstructions and common experiments in perception research.

In an eye tracking experiment, the digital reconstructions were each presented for 6 s among other similar Medieval Christian tempera paintings. Faces attracted gaze quickly and maintained most of the gaze (>50% of total looking time). No differences were observed for either face-gaze measure between the two versions of the Lindau Crucifixion. Angels were looked at less than the faces overall, but no differences were observed in total looking time to the angels between the two versions. The time to first look at any angel was similar across the two versions, at least for participants that fixated an angel in both versions of the Lindau Crucifixion. Yet, for the blue background, fewer participants fixated an angel at all, with an apparently higher time for 50% of the participants to fixate any angel. Thus, gaze behavior was very similar overall across the two versions of the Lindau Crucifixion. However, when considering both the number of participants that did or did not fixate any angel together with the time to fixate an angel, some differences were observed suggesting that the angels may attract gaze slightly less against the blue background than the gold background.

In the gist perception experiment the digital reconstructions were each presented for 2.5 s. Here, we observed that the same visual elements were mentioned by participants, regardless of the background color, mainly focusing on the three central figures in the painting, their appearance and behavior.

Our findings align with the notion that human faces attract and maintain attention or gaze (e.g., Bindemann et al., 2005; Birmingham et al., 2008a, 2008b). Moreover, previous eye-tracking work on artworks has shown that while viewers spend a few seconds on a painting, they fixate primarily on salient features such as human figures or faces (Villani et al., 2015). We see this bias reflected in both the gaze measures and in the textual responses, and conclude that during the brief viewing, participants consistently attended to figures and their behaviors, regardless of the manipulated background.

Thus, no major differences in gaze allocation and perception were established between the two versions of the Lindau Crucifixion on this timescale, with these visual representations. Regarding the representativeness of the timescale, it has been reported that the mean amount of time museum visitors spend looking at an artwork is roughly 27 s, with a median and mode of 17 and 10 s, respectively (Smith & Smith, 2001; Smith et al., 2017). The 6 s presentation in the eye-tracking experiment thus captures most of the gaze allocation that may be observed in a museum context. For extended viewing durations, that is, under scrutiny and potential interpretation of the artwork, differences may still be observed. However, we also expect that these would be related to the expertise of the viewer with the painting style and period, or Christian symbolism.

Regarding the visual presentations, we cannot rule out that relying on the digital versions instead of physical artworks or facsimiles impacted gaze allocation or perception at a glance. For example, the digital reconstruction fails to capture the spectral reflectance properties of the gilded background, which may impact the subjective qualities of an artwork (Carbon & Deininger, 2013) or have subtle effects on gaze allocation (Leonards et al., 2007). However, each change in experimental set-up has methodological consequences. Using physical artworks instead of digital reconstructions for an experiment poses several practical challenges. For example, it becomes more difficult to control the duration and onset of the artwork. Second, selecting unambiguous task instructions and checking adherence to them is not trivial, making it more complicated to obscure the study's focus from the participants. Finally, it would require a move from screen-based to wearable eye trackers with substantial consequences for (automated) data analysis (see, e.g., Hessels et al., 2020). The existing facsimiles of the Lindau Crucifixion may allow us to investigate the generalization of our present findings to the physical artwork in the future.

In conclusion, we observed no differences in gist perception and only minor differences in gaze allocation between two versions of the Lindau Crucifixion under brief viewing conditions. How might these findings, or the methods we employed, impact art restoration decisions? A central principle in art conservation is the avoidance of irreversible interventions. In the case of the Lindau Crucifixion, the painting has been overpainted, while restoration ethics preclude a full reconstruction of an earlier state. As a result, it is not possible to directly assess whether the decision not to restore the work affects the perception of, for example, its original iconographical and liturgical meaning. Our study addressed this limitation by investigating the consequences of different conservation choices through controlled, hypothetical alterations. By simulating alternative versions of an artwork, such experiments allow conservators and art historians to examine how gaze behavior and overall perception may change, something that cannot be studied on the physical object itself. The empirical data not only make it possible to assess the perceptual consequences of restoration decisions that were previously inaccessible, but may, in the longer term, inform broader discussions on conservation practice by complementing historical and ethical considerations with data on observer behavior.

## Supplemental Material

sj-pdf-1-pec-10.1177_03010066261433859 - Supplemental material for Blue or gold? Visual perception and the restoration of a Medieval painting Supplemental material, sj-pdf-1-pec-10.1177_03010066261433859 for Blue or gold? Visual perception and the restoration of a Medieval painting by Feline A. van Gelderen, Yuexi Zhu, Ignace T.C. Hooge, Sanne Frequin and Roy S. Hessels in Perception

## References

[bibr1-03010066261433859] Anwyl-Irvine A. L. Massonnié J. Flitton A. Kirkham N. Evershed J. K. (2020). Gorilla in our midst: An online behavioral experiment builder. Behavior Research Methods, 52, 388–407. 10.3758/s13428-019-01237-x 31016684 PMC7005094

[bibr2-03010066261433859] Bindemann M. Burton A. M. Hooge I. T. C. Jenkins R. De Haan E. H. F. (2005). Faces retain attention. Psychonomic Bulletin & Review, 12(6), 1048–1053. 10.3758/bf03206442 16615327

[bibr3-03010066261433859] Birmingham E. Bischof W. F. Kingstone A. (2008a). Social attention and real-world scenes: The roles of action, competition and social content. The Quarterly Journal of Experimental Psychology, 61(7), 986–998. 10.1080/17470210701410375 18938281

[bibr4-03010066261433859] Birmingham E. Bischof W. F. Kingstone A. (2008b). Gaze selection in complex social scenes. Visual Cognition, 16(2–3), 341–355. 10.1080/13506280701434532

[bibr5-03010066261433859] Carbon C. Deininger P. (2013). Golden perception: Simulating perceptual habits of the past. i-Perception, 4(6), 468–476. 10.1068/i0605 24349703 PMC3859561

[bibr7-03010066261433859] Fei-Fei L. Iyer A. Koch C. Perona P. (2007). What do we perceive in a glance of a real-world scene? Journal of Vision, 7(1), 1–29. 10.1167/7.1.10 17461678

[bibr8-03010066261433859] Hayes A. F. Krippendorff K. (2007). Answering the call for a standard reliability measure for coding data. Communication Methods and Measures, 1(1), 77–89. 10.1080/19312450709336664

[bibr9-03010066261433859] Hessels R. S. Benjamins J. S. van Doorn A. J. Koenderink J. J. Hooge I. T. C. (2021). Perception of the potential for interaction in social scenes. i-Perception, 12(5), 1–25. 10.1177/20416695211040237 PMC8474344 34589197

[bibr10-03010066261433859] Hessels R. S. Kemner C. Van Den Boomen C. Hooge I. T. C. (2016). The area-of-interest problem in eyetracking research: A noise-robust solution for face and sparse stimuli. Behavior Research Methods, 48(4), 1694–1712. 10.3758/s13428-015-0676-y 26563395 PMC5101255

[bibr11-03010066261433859] Hessels R. S. Niehorster D. C. Holleman G. A. Benjamins J. S. Hooge I. T. C. (2020). Wearable technology for “real-world research”: Realistic or not? Perception, 49(6), 611–615. 10.1177/0301006620928324 32552490 PMC7307000

[bibr12-03010066261433859] Hessels R. S. Niehorster D. C. Kemner C. Hooge I. T. C. (2017). Noise-robust fixation detection in eye movement data: Identification by two-means clustering (I2MC). Behavior Research Methods, 49(5), 1802–1823. 10.3758/s13428-016-0822-1 27800582 PMC5628191

[bibr13-03010066261433859] Hooge I. Camps G. (2013). Scan path entropy and arrow plots: Capturing scanning behavior of multiple observers. Frontiers in Psychology, 4, 996. 10.3389/fpsyg.2013.00996 24399993 PMC3872074

[bibr14-03010066261433859] Hooge I. T. C. Nuthmann A. Nyström M. Niehorster D. C. Holleman G. A. Andersson R. Hessels R. S. (2025). The fundamentals of eye tracking part 2: From research question to operationalization. Behavior Research Methods, 57(2). 10.3758/s13428-024-02590-2 PMC11761893 39856471

[bibr15-03010066261433859] Hoogerbrugge A. J. Hooge I. T. C. Hessels R. S. Strauch C. (2025). When is enough enough? Empirical guidelines to determine participant sample size for scene viewing studies. Behavior Research Methods, 57(9), 241. 10.3758/s13428-025-02754-8 40721900 PMC12304073

[bibr16-03010066261433859] Itti L. Koch C. (2000). A saliency-based search mechanism for overt and covert shifts of visual attention. Vision Research, 40(10–12), 1489–1506. 10.1016/S0042-6989(99)00163-7 10788654

[bibr17-03010066261433859] JASP Team. (2024). JASP (Version 0.19.3) [Computer software].

[bibr18-03010066261433859] Kim D. Y. (2019). Points on a field: Gentile da Fabriano and gold ground. Journal of Early Modern History, 23(2–3), 191–226. 10.1163/15700658-12342636

[bibr19-03010066261433859] Langton S. R. Law A. S. Burton A. M. Schweinberger S. R. (2008). Attention capture by faces. Cognition, 107(1), 330–342. 10.1016/j.cognition.2007.07.012 17767926

[bibr20-03010066261433859] Leonards U. Baddeley R. Gilchrist I. D. Troscianko T. Ledda P. Williamson B. (2007). Mediaeval artists: Masters in directing the observers’ gaze. Current Biology, 17(1), R8–R9. 10.1016/j.cub.2006.11.046 17208178

[bibr21-03010066261433859] Locher P. Krupinski E. A. Mello-Thoms C. Nodine C. F. (2007). Visual interest in pictorial art during an aesthetic experience. Spatial Vision, 21(1), 55–77. 10.1163/156856808782713762 18073051

[bibr22-03010066261433859] Muñoz Viñas S. (2005). Contemporary theory of conservation. Elsevier Ltd.

[bibr23-03010066261433859] Oliva A. (2005). Gist of the scene. In Neurobiology of attention (pp. 251–256). Academic Press.

[bibr24-03010066261433859] Schönbrodt F. D. Wagenmakers E. (2018). Bayes Factor design analysis: Planning for compelling evidence. Psychonomic Bulletin & Review, 25(1), 128–142. 10.3758/s13423-017-1230-y 28251595

[bibr25-03010066261433859] Smith J. K. Smith L. F. (2001). Spending time on art. Empirical Studies of the Arts, 19(2), 229–236. 10.2190/5mqm-59jh-x21r-jn5j

[bibr26-03010066261433859] Smith L. F. Smith J. K. Tinio P. P. L. (2017). Time spent viewing art and reading labels. Psychology of Aesthetics Creativity and the Arts, 11(1), 77–85. 10.1037/aca0000049

[bibr29-03010066261433859] Theeuwes J. Van Der Stigchel S. (2006). Faces capture attention: Evidence from inhibition of return. Visual Cognition, 13(6), 657–665. 10.1080/13506280500410949

[bibr30-03010066261433859] Thorpe S. Fize D. Marlot C. (1996). Speed of processing in the human visual system. Nature, 381, 520–522. 10.1038/381520a0 8632824

[bibr31-03010066261433859] Tissen L. N. M. Frequin S. Wiersma R. T. (2023). The case of the golden background, a virtual restoration and a physical reconstruction of the medieval crucifixion of the Lindau Master (C. 1425). Digital Humanities Quarterly, 17(1), 1.

[bibr32-03010066261433859] Villani D. Morganti F. Cipresso P. Ruggi S. Riva G. Gilli G. (2015). Visual exploration patterns of human figures in action: an eye tracker study with art paintings. Frontiers in Psychology, 6, 10.3389/fpsyg.2015.01636 PMC4620395 26579021

