# Supplementary material for: Blue or gold? Visual perception and the restoration of a Medieval painting
Source: Perception. 2026 Mar 29;55(9):819–31. doi: 10.1177/03010066261433859 (PMC13494132; doi:10.1177/03010066261433859)
Supplement: Supplementary file 1 [file sj-pdf-1-pec-10.1177_03010066261433859.pdf]

Supplementary material - Artworks used as stimuli

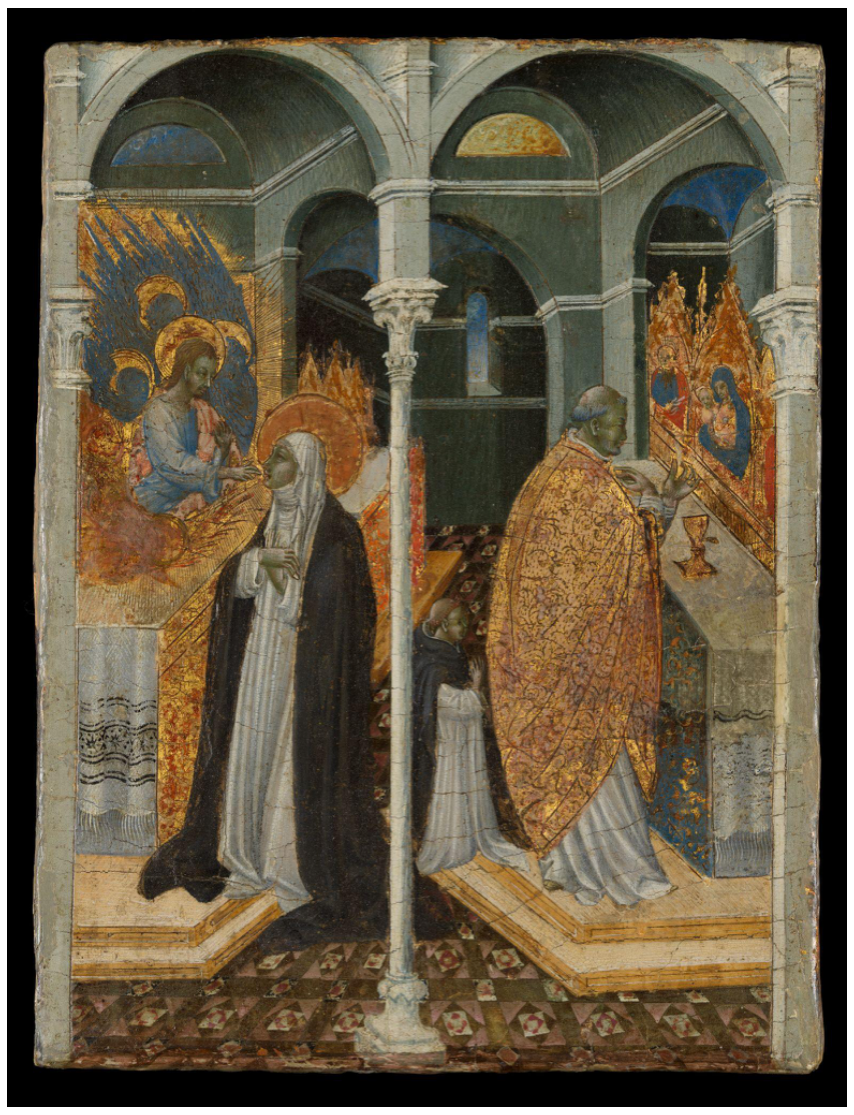

Title: The Miraculous Communion of Saint Catherine of Siena
Author: Giovanni di Paolo (Giovanni di Paolo di Grazia)
Museum:The Metropolitan Museum of Art, New York
Inventory number: 32.100.95
Date: 1461
Technique: Tempera and gold
Support: Wood
Dimension: 28.9 x 22.2 cm
Source: The Miraculous Communion of Saint Catherine of Siena | The Metropolitan Museum of Art

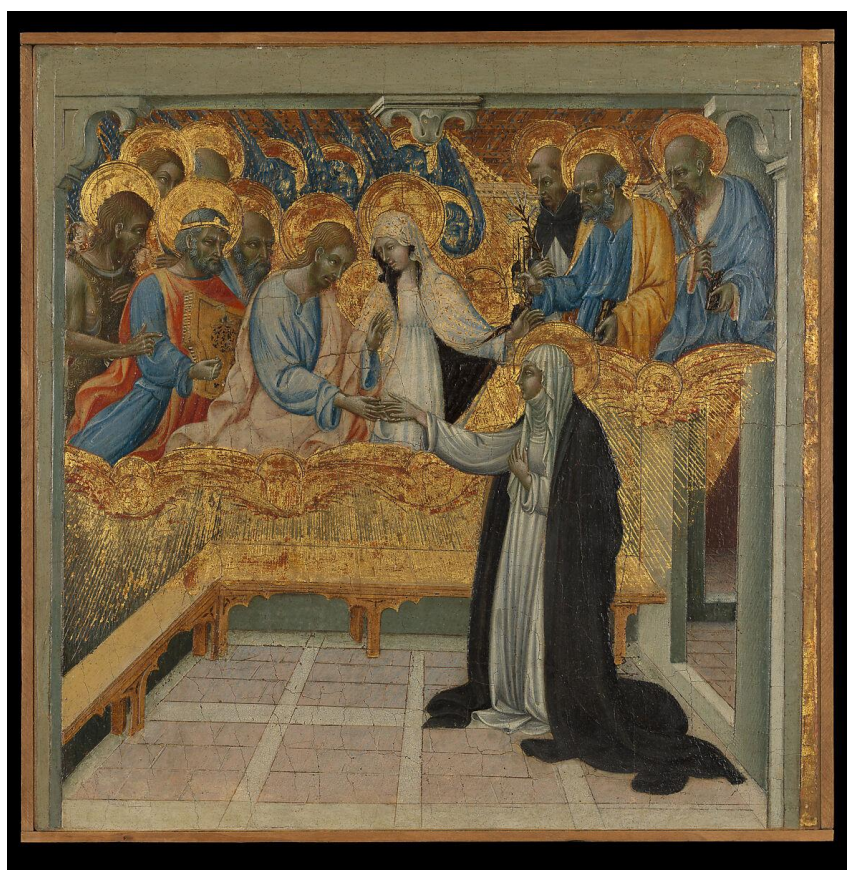

Title: The Mystic Marriage of Saint Catherine of Siena
Author: Giovanni di Paolo (Giovanni di Paolo di Grazia)
Museum:The Metropolitan Museum of Art, New York
Inventory number: 1997.117.2
Date: 1460
Technique: Tempera and gold
Support: Wood
Dimension: 28.9 x 28.9 cm
Source: The Mystic Marriage of Saint Catherine of Siena | The Metropolitan Museum of Art

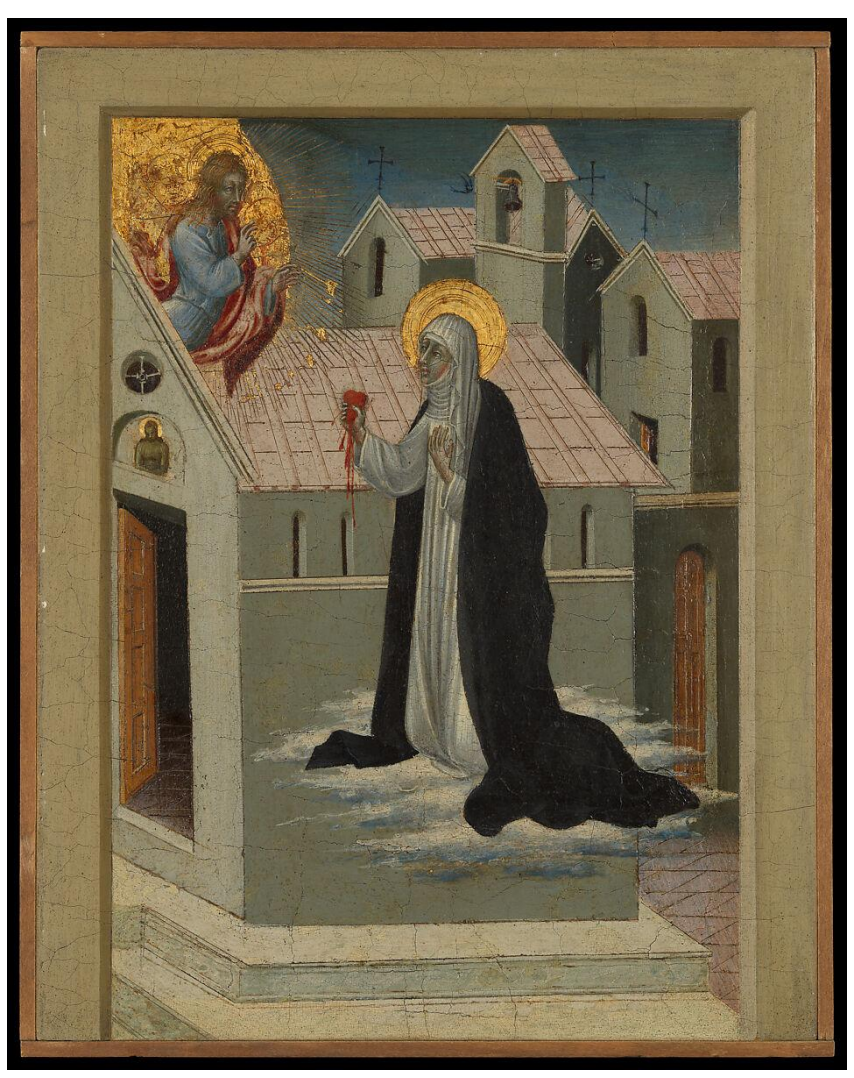

Title: Saint Catherine of Siena Exchanging Her Heart with Christ
Author: Giovanni di Paolo (Giovanni di Paolo di Grazia)
Museum:The Metropolitan Museum of Art, New York
Inventory number: 1997.117.3
Date: 1461
Technique: Tempera and gold
Support: Wood
Dimension: 28.9 x 22.5 cm
Source: Saint Catherine of Siena Exchanging Her Heart with Christ | The Metropolitan Museum of Art

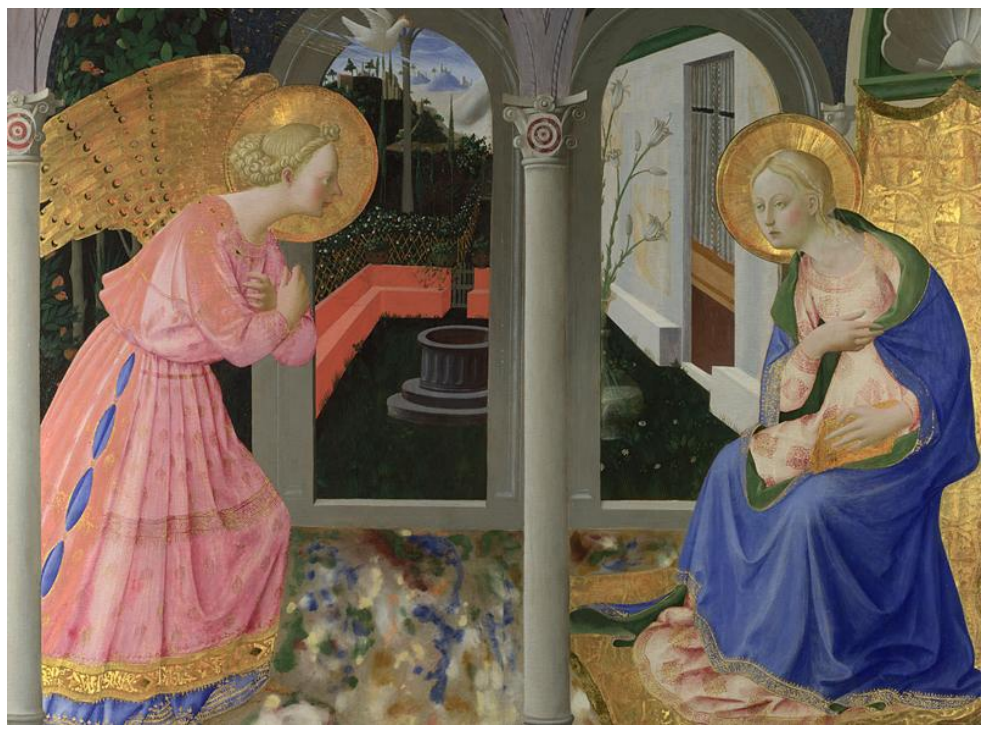

Title: The Annunciation
Author: Zanobi Strozzi
Museum: The National Gallery, London
Inventory number: NG1406
Date: ca. 1440-5
Technique: Egg tempera
Support: Wood panel
Dimension: 104.5 × 142 cm
Source: Zanobi Strozzi | The Annunciation | NG1406 | National Gallery, London

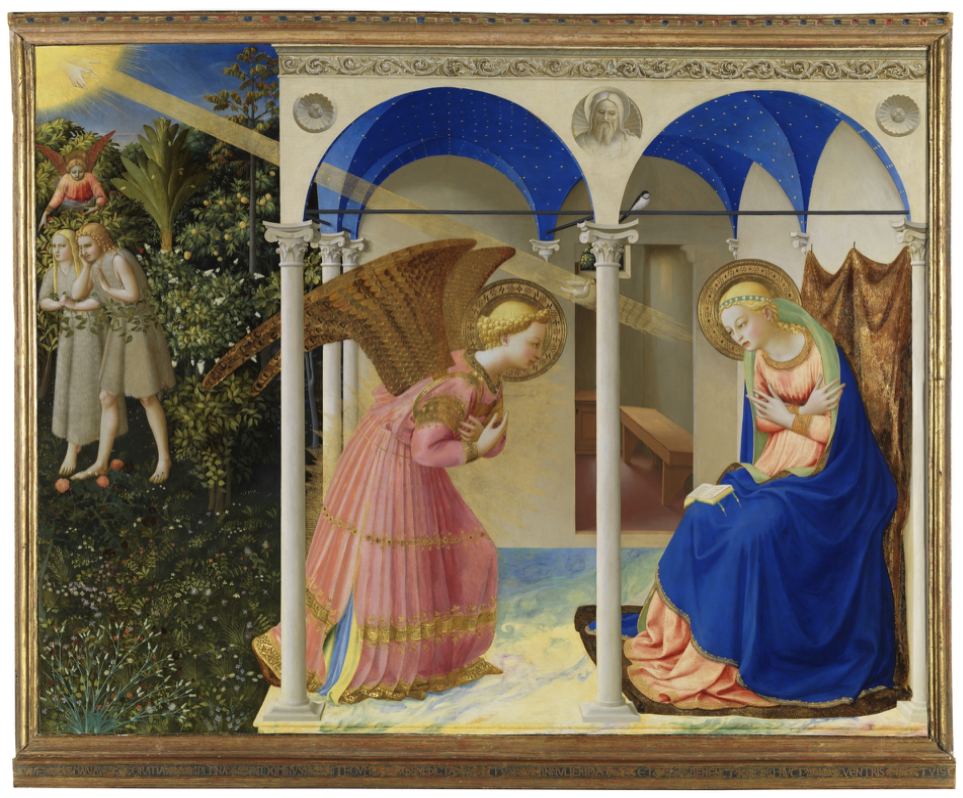

Title: The Annunciation
Author: Angelico Fra
Museum: Prado Museum, Madrid
Inventory number: P000015
Date: 1425 - 1426
Technique: Egg tempera
Support: Poplar panel
Dimension: Height: 190.3 cm; Width: 191.5 cm
Source: The Annunciation

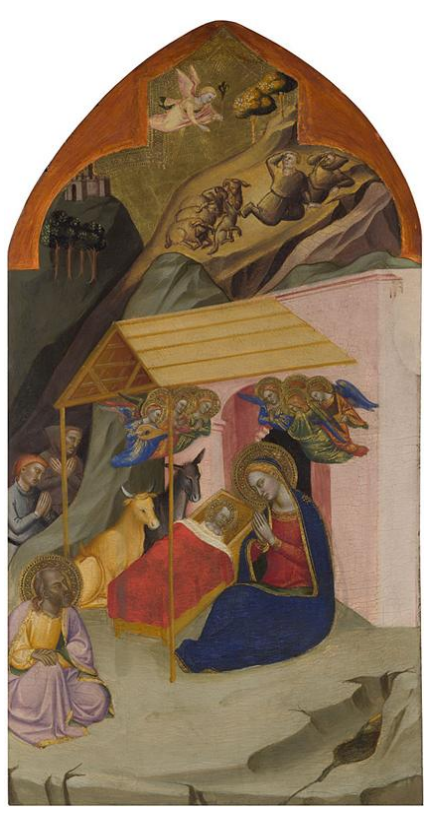

Title: The Nativity with the Annunciation to the Shepherds
Author: Jacopo di Cione and workshop
Museum: The National Gallery, London
Inventory number: NG573
Date: 1370-1371
Technique: Egg tempera
Support: Wood panel
Dimension: 95.5 × 49.4 cm
Source: Jacopo di Cione and workshop | The Nativity with the Annunciation to the Shepherds | NG573 | National Gallery, London

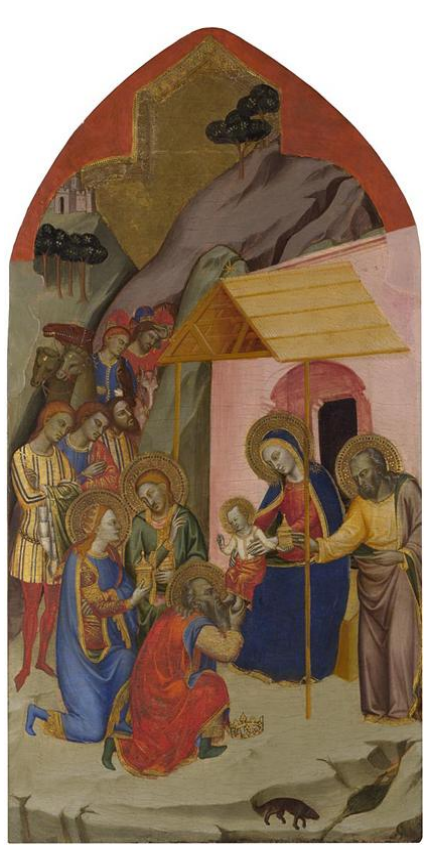

Title: The Adoration of the Kings: Upper Tier Panel
Author: Jacopo di Cione and workshop
Museum: The National Gallery, London
Inventory number: NG574
Date: 1370-1371
Technique: Egg tempera
Support: Wood panel
Dimension: 95.5 × 49.3 cm
Source: Jacopo di Cione and workshop | The Adoration of the Kings: Upper Tier Panel | NG574 | National Gallery, London

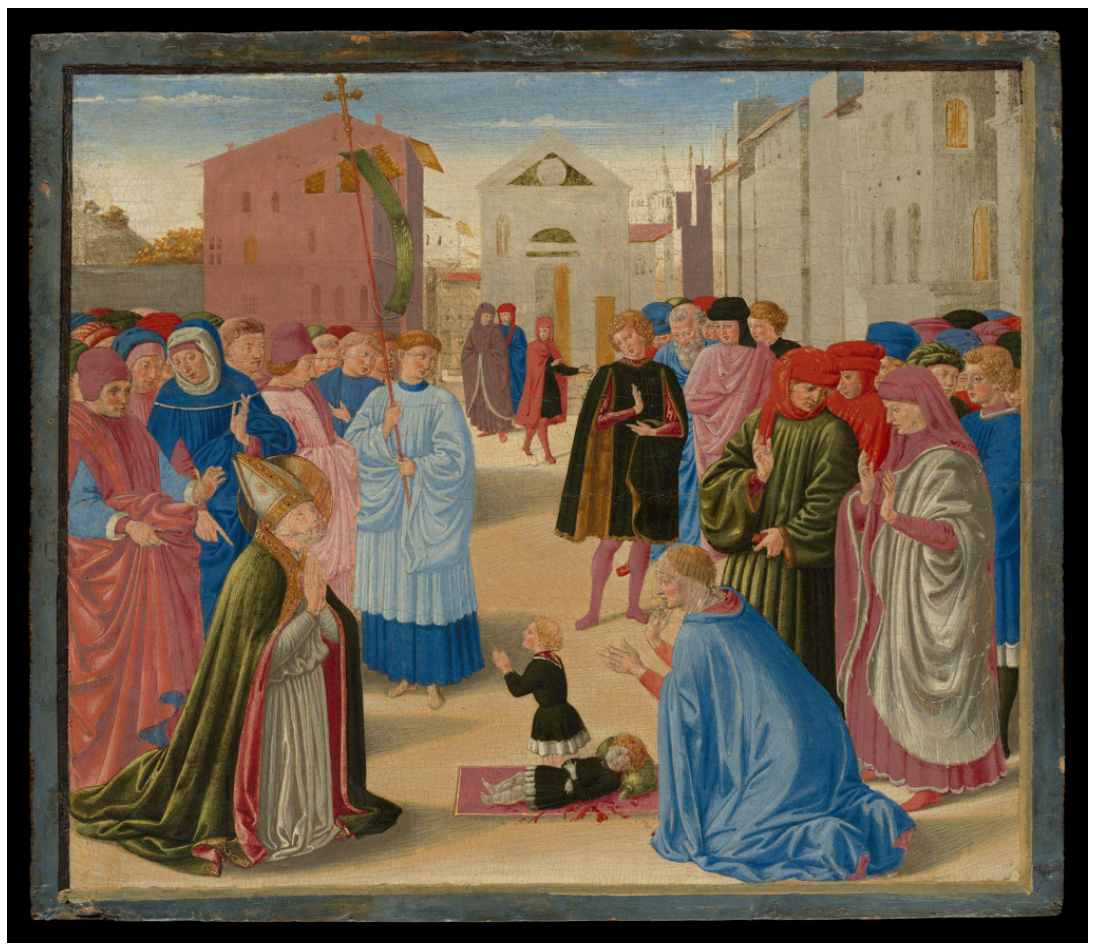

Title: Saint Zenobius Resuscitating a Dead Child
Author: Benozzo Gozzoli (Benozzo di Lese di Sandro)
Museum: The Metropolitan Museum of Art, New York
Inventory number: 15.106.3
Date: ca. 1420–1497
Technique: Egg tempera
Support: Wood panel
Dimension: 39.4 x 45.7 cm
Source: Benozzo Gozzoli (Benozzo di Lese di Sandro) | Saint Zenobius Resuscitating a Dead Child | The Metropolitan Museum of Art

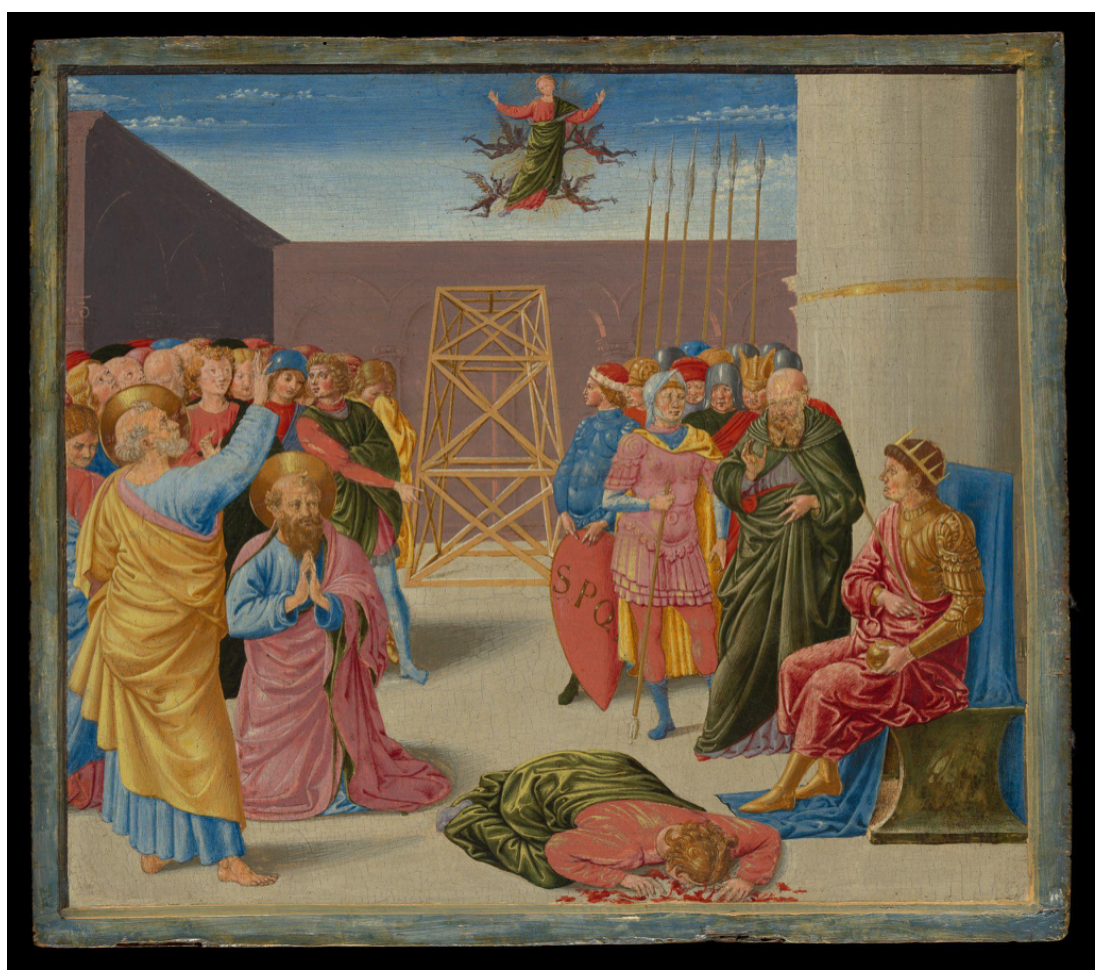

Title: Saint Peter and Simon Magus
Author: Benozzo Gozzoli (Benozzo di Lese di Sandro)
Museum: The Metropolitan Museum of Art, New York
Inventory number: 15.106.1
Date: ca. 1420–1497
Technique: Egg tempera
Support: Wood panel
Dimension: 40 x 45.7 cm
Source: Benozzo Gozzoli (Benozzo di Lese di Sandro) | Saint Peter and Simon Magus | The Metropolitan Museum of Art

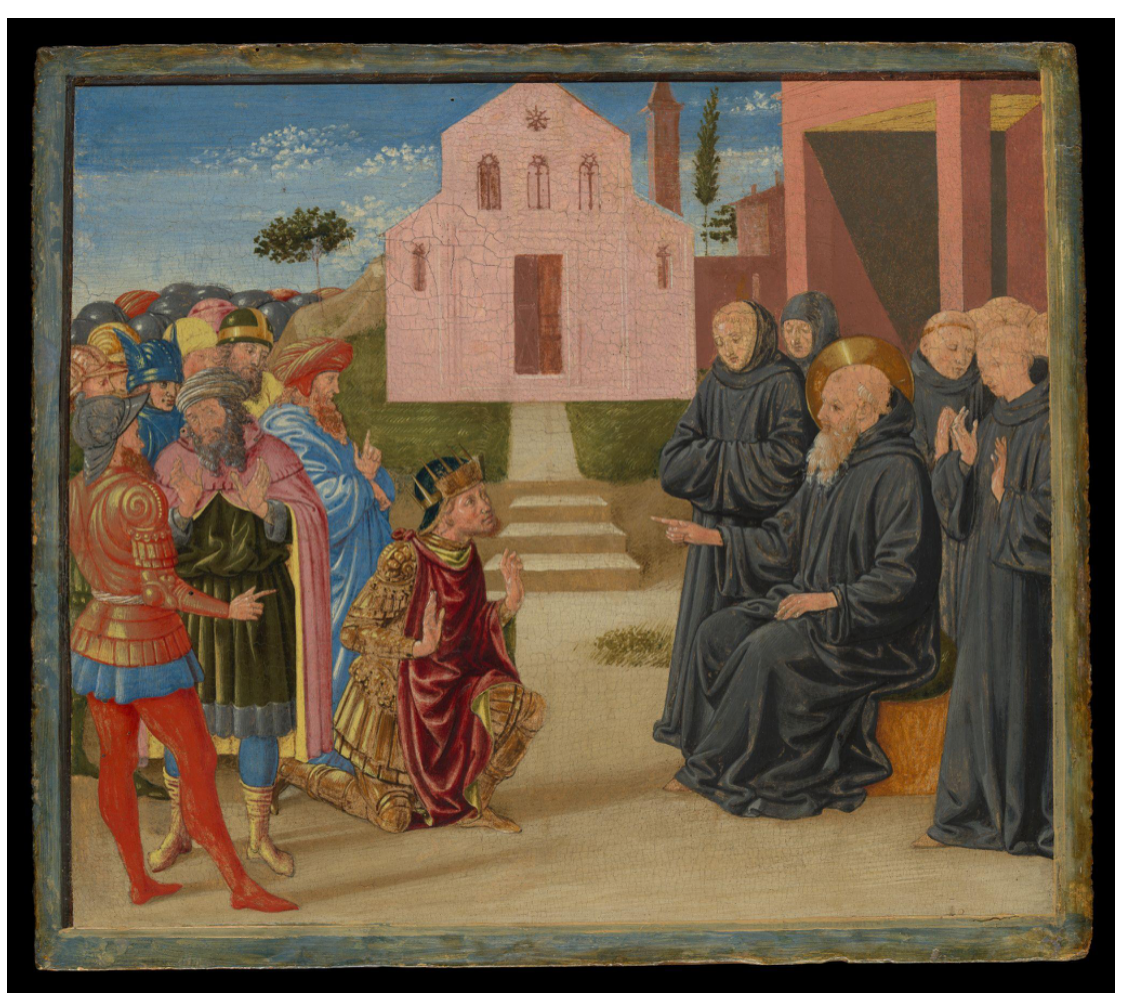

Title:Totila before Saint Benedict
Author: Benozzo Gozzoli (Benozzo di Lese di Sandro)
Museum: The Metropolitan Museum of Art, New York
Inventory number: 15.106.4
Date: ca. 1420–1497
Technique: Egg tempera
Support: Wood panel
Dimension: 39.4 x 45.7 cm
Source: Benozzo Gozzoli (Benozzo di Lese di Sandro) | Totila before Saint Benedict | The Metropolitan Museum of Art

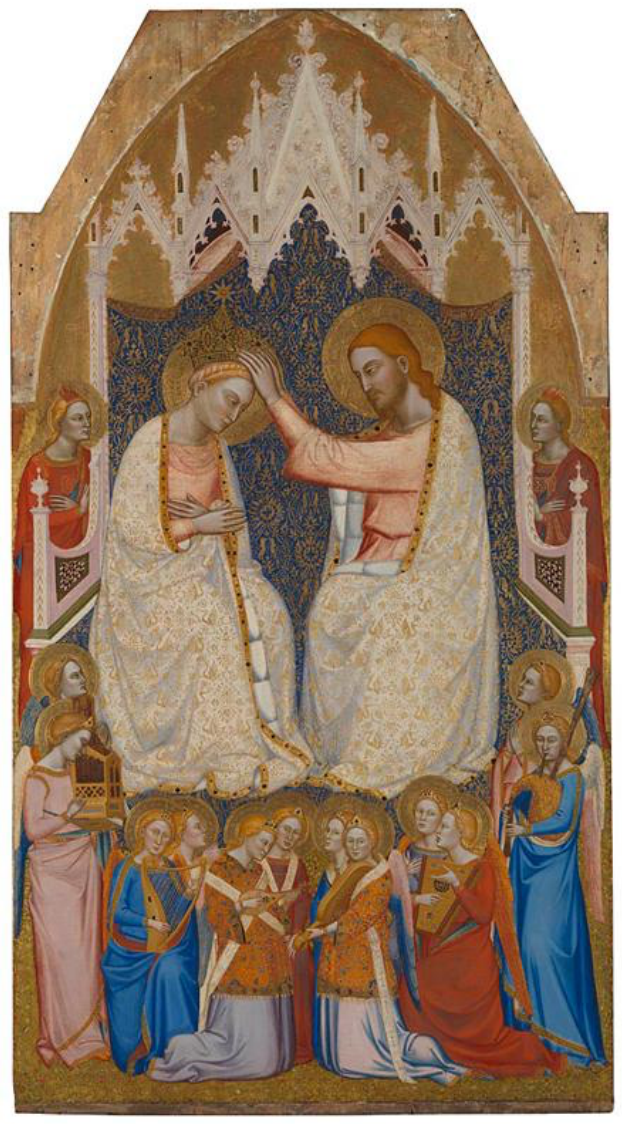

Title: The Coronation of the Virgin: Central Main Tier Panel
Author: Jacopo di Cione and workshop
Museum: The National Gallery, London
Inventory number: NG569.1
Date: ca. 1370-1
Technique: Egg tempera
Support: Wood panel
Dimension: 206.5 × 113.5 cm
Source:
[Jacopo di Cione and workshop | The Coronation of the Virgin: Central Main Tier Panel | NG569.1 | National Gallery, London]

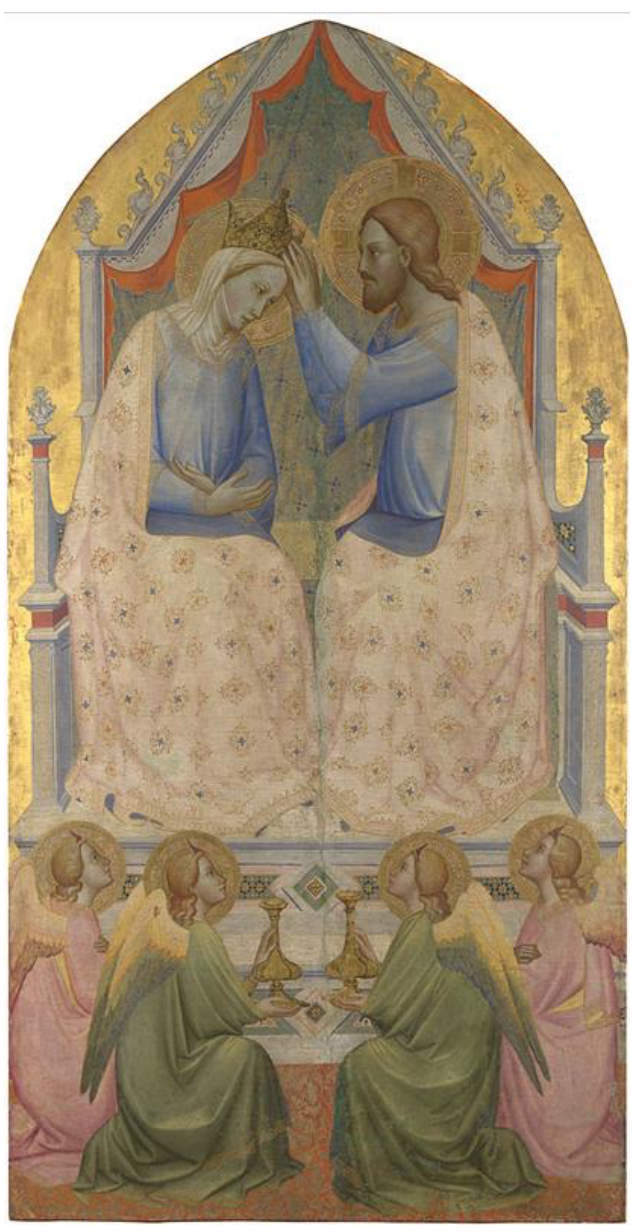

Title: The Coronation of the Virgin
Author: Agnolo Gaddi
Museum: The National Gallery, London
Inventory number: NG568
Date: ca. 1380-85
Technique: Egg tempera
Support: Wood panel
Dimension: 183.6 × 94.3 cm
Source: [Agnolo Gaddi | The Coronation of the Virgin | NG568 | National Gallery, London]

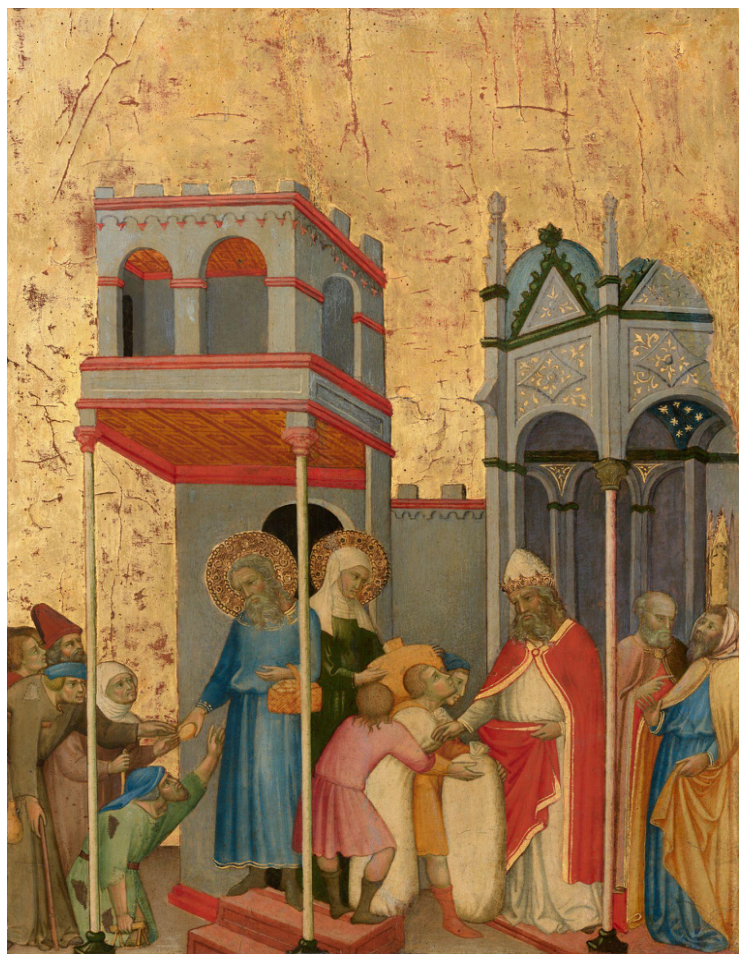

Title: Joachim and Anna Giving Food to the Poor and Offerings to the Temple
Author: Andrea di Bartolo
Museum: National Gallery of Art, Washington DC
Inventory number: 1939.1.43
Date: c. 1400/1405
Technique: Tempera
Support: Poplar panel
Dimension: 44.1 × 32.5 cm
Source: Joachim and Anna Giving Food to the Poor and Offerings to the Temple by Andrea di Bartolo

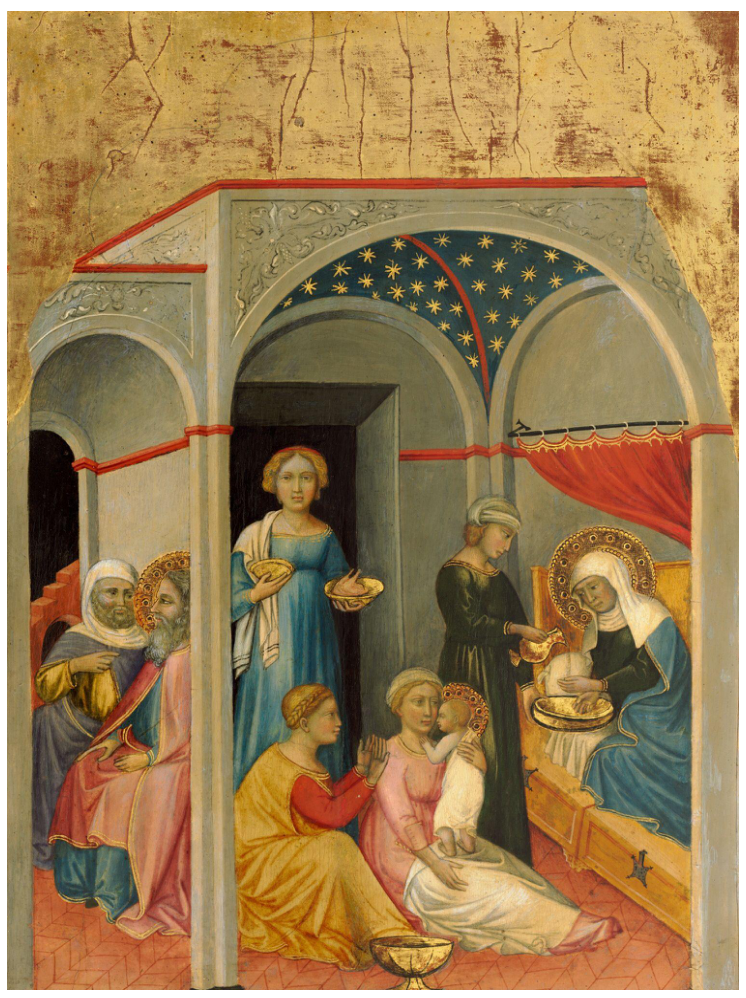

Title: The Nativity of the Virgin
Author: Andrea di Bartolo
Museum: National Gallery of Art, Washington DC
Inventory number: 1939.1.42
Date: c. 1400/1405
Technique: Tempera
Support: Poplar panel
Dimension: 44.2 × 32.5 cm
Source: The Nativity of the Virgin by Andrea di Bartolo

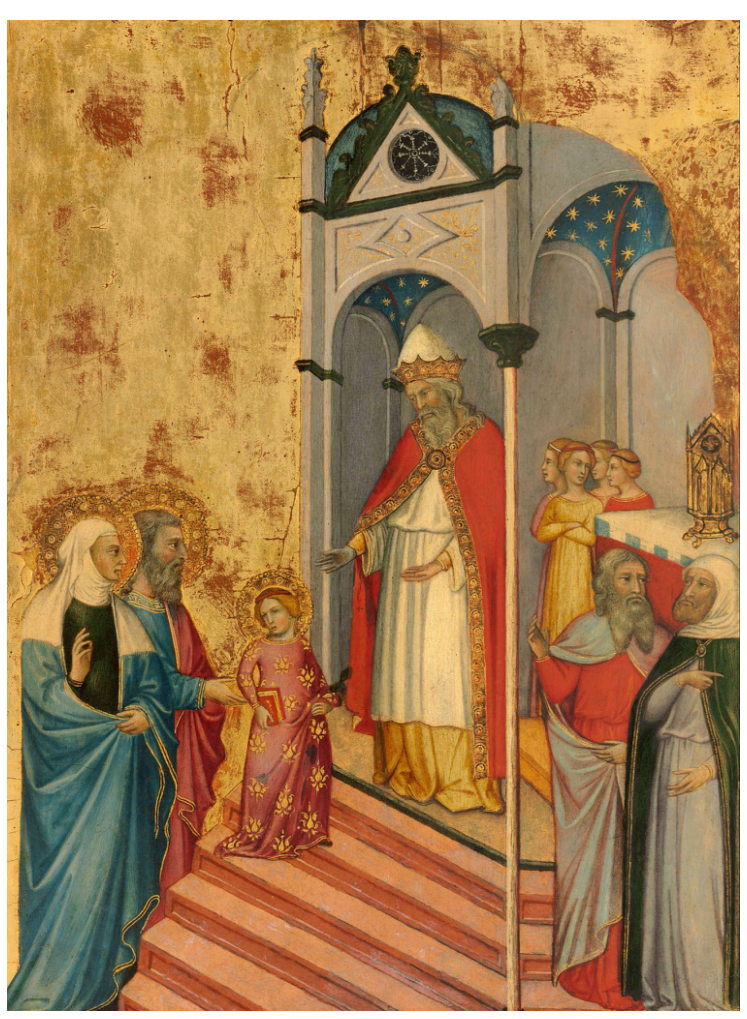

Title: The Presentation of the Virgin in the Temple
Author: Andrea di Bartolo
Museum: National Gallery of Art, Washington DC
Inventory number: 1939.1.41
Date: c. 1400/1405
Technique: Tempera
Support: Poplar panel
Dimension: 44 × 32.3 cm
Source: The Presentation of the Virgin in the Temple by Andrea di Bartolo

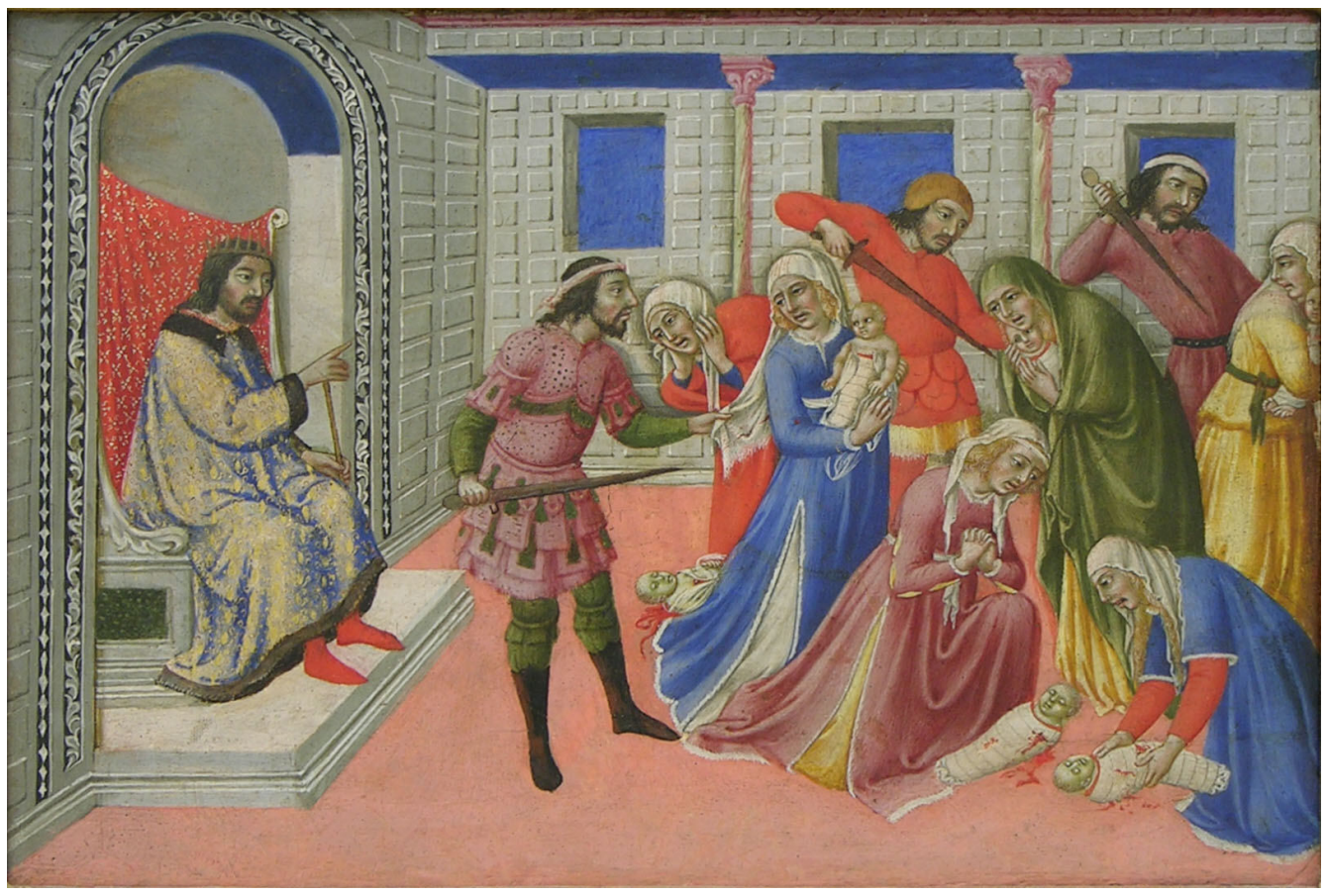

Title: The Massacre of the Innocents
Author: Sano di Pietro (Ansano di Pietro di Mencio)
Museum: The Metropolitan Museum of Art, New York
Inventory number: 58.189.1
Date: ca. 1470
Technique: Tempera
Support: Wood
Dimension: 30.2 x 44.1 cm
Source: Sano di Pietro (Ansano di Pietro di Mencio) | The Massacre of the Innocents | The Metropolitan Museum of Art

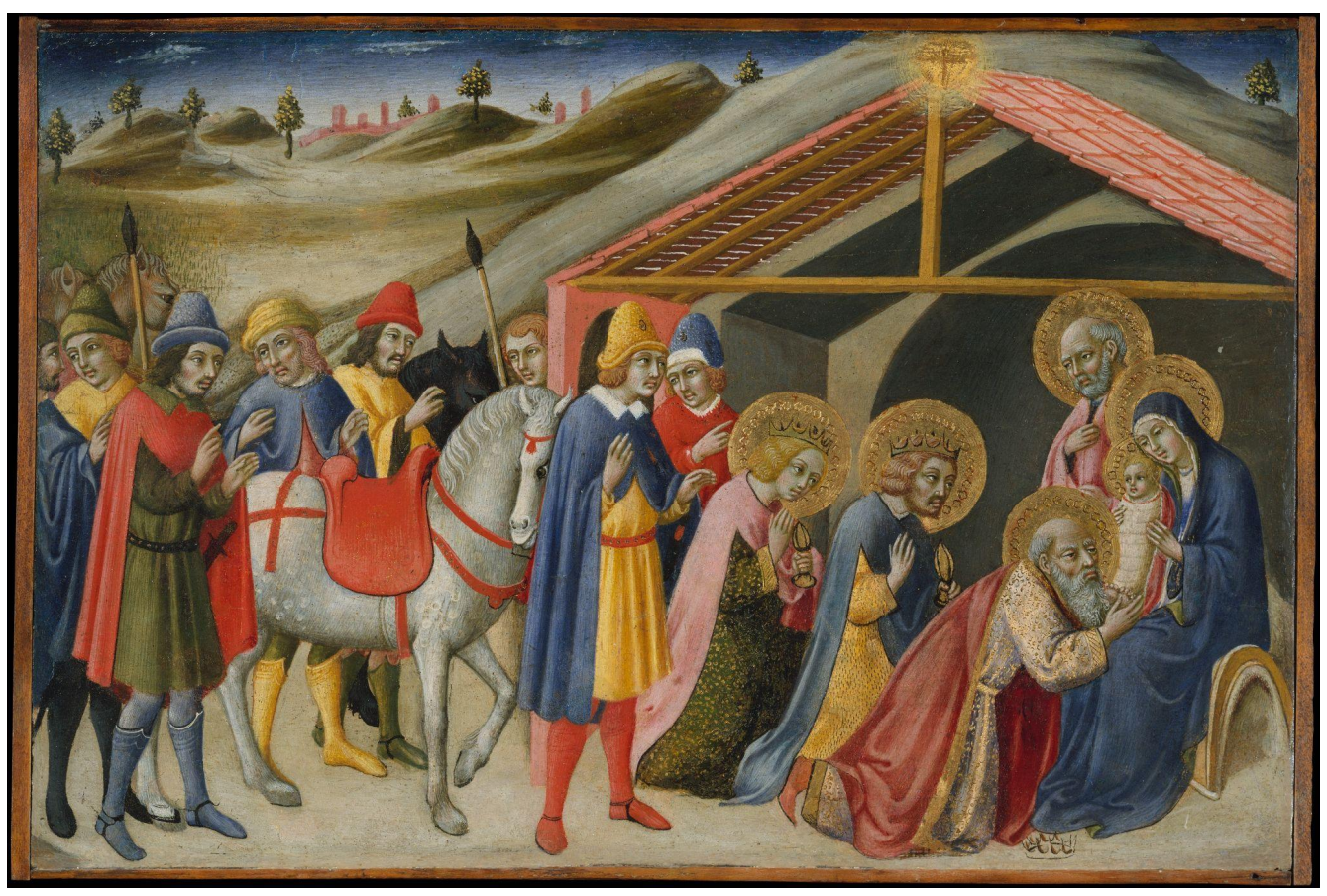

Title: The Adoration of the Magi
Author: Sano di Pietro (Ansano di Pietro di Mencio)
Museum: The Metropolitan Museum of Art, New York
Inventory number: 58.189.2
Date: ca. 1470
Technique: Tempera and gold
Support: Wood
Dimension: 30.2 x 47.6 cm
Source: The Adoration of the Magi

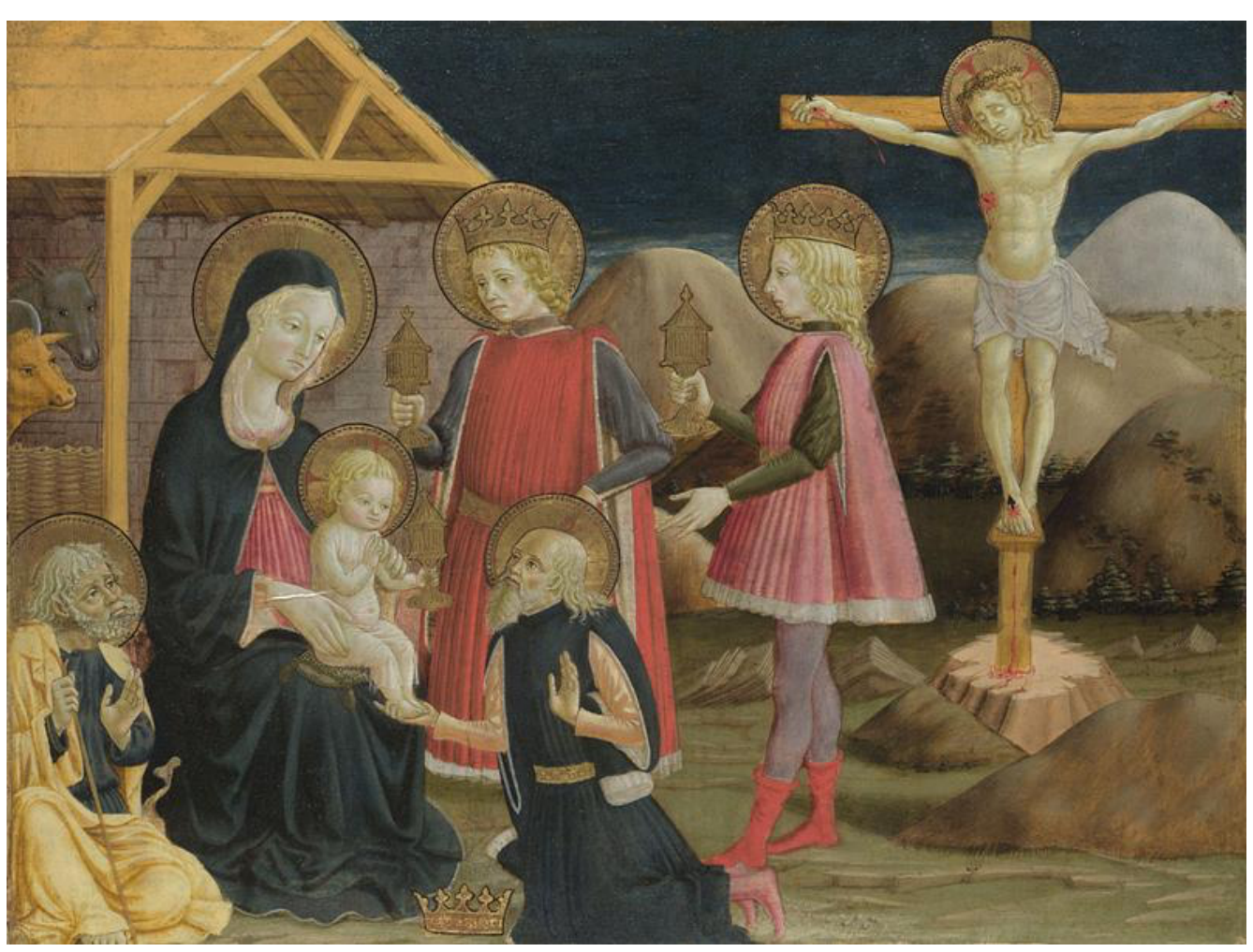

Title: The Adoration of the Kings, and Christ on the Cross
Author: Probably by Benedetto Bonfigli
Museum: The National Gallery, London
Inventory number: NG1843
Date: ca. 1465-75
Technique: Egg tempera
Support: Wood panel
Dimension: 37.5 × 49.5 cm
Source: Probably by Benedetto Bonfigli | The Adoration of the Kings, and Christ on the Cross | NG1843 | National Gallery, London

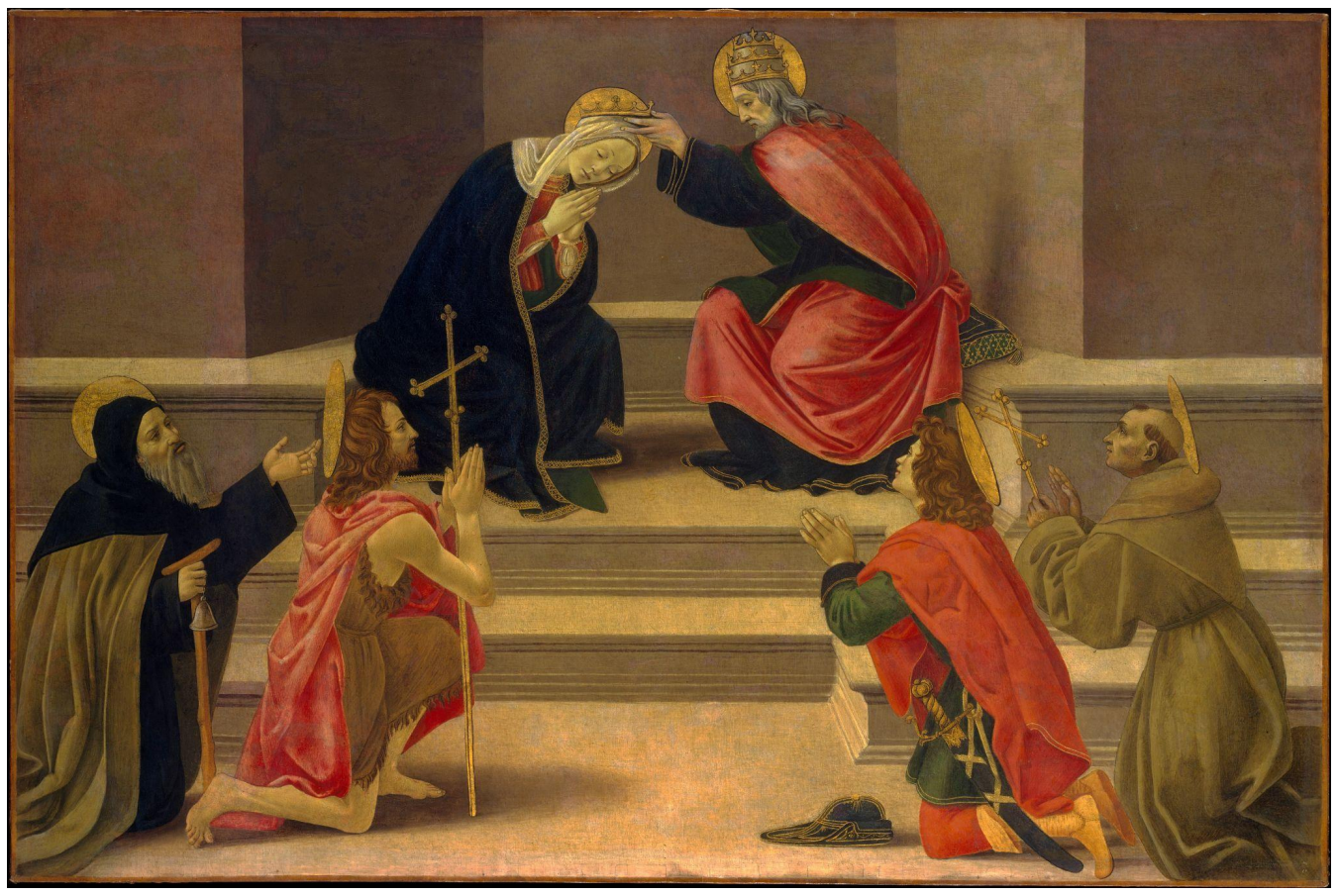

Title: The Coronation of the Virgin
Author: Follower of Botticelli
Museum: The Metropolitan Museum of Art, New York
Inventory number: 49.7.4
Date: Fourth quarter 15th century
Technique: Tempera
Support: Canvas, transferred from wood
Dimension: 100.3 x 153 cm
Source: [Follower of Botticelli | The Coronation of the Virgin | The Metropolitan Museum of Art]

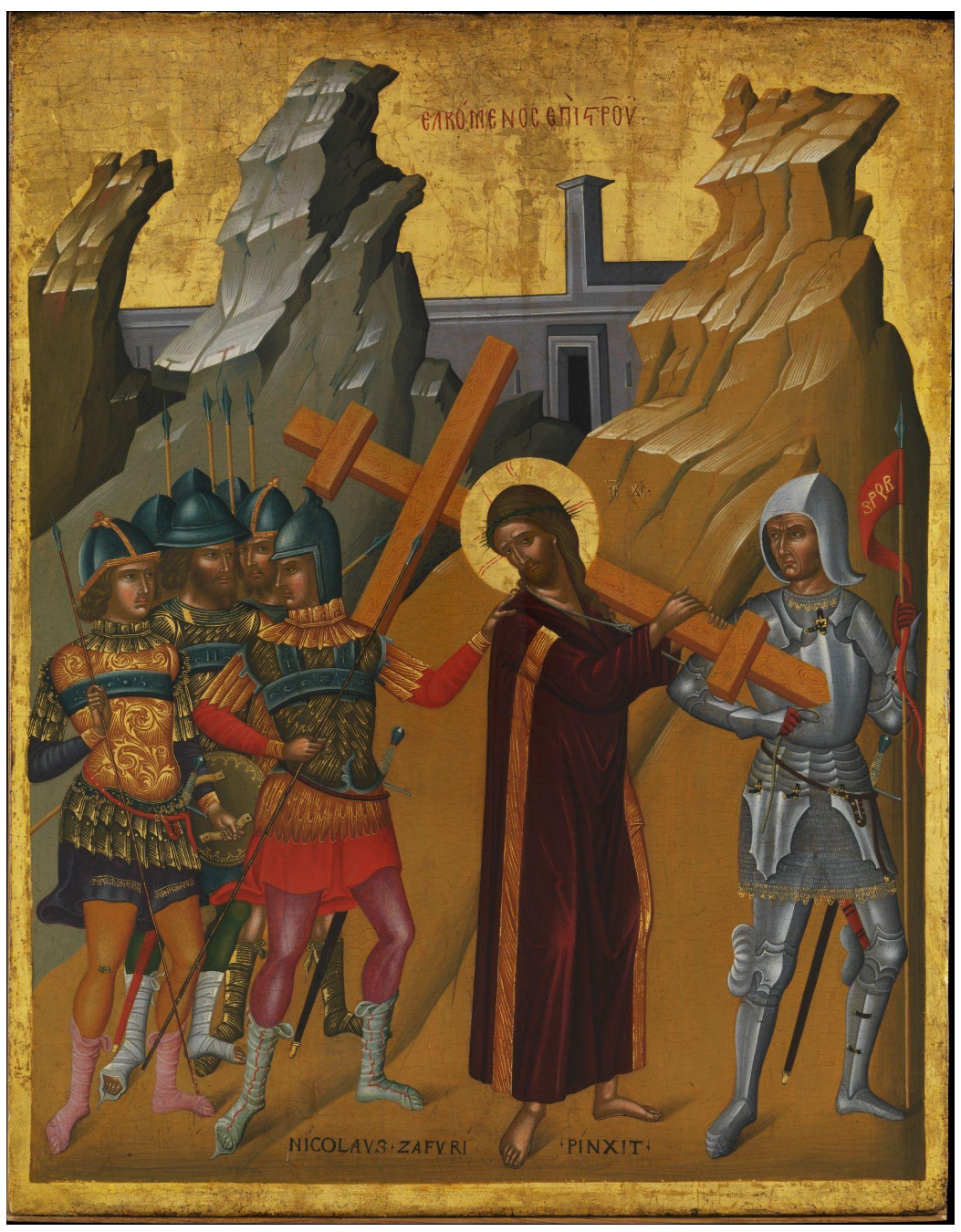

Title: Christ Bearing the Cross
Author: Nicolaos Tzafouris
Museum: The Metropolitan Museum of Art, New York
Inventory number: 29.158.746
Date: ca. 1455–1500/1501
Technique: Oil and tempera
Support: Wood, gold ground
Dimension: 69.2 x 54.6 cm
Source: [Nicolaos Tzafouris | Christ Bearing the Cross | The Metropolitan Museum of Art]

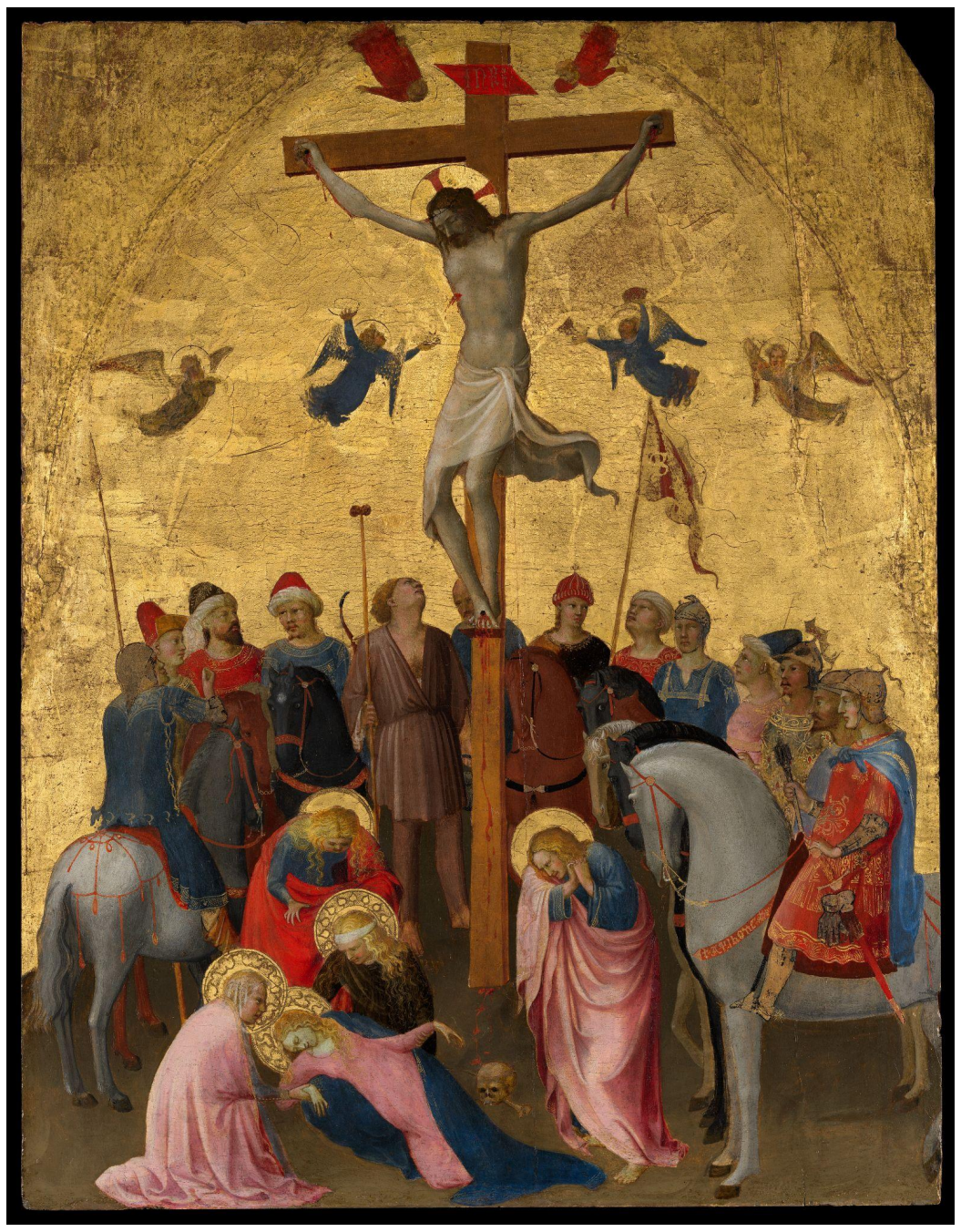

Title: The Crucifixion
Author: Fra Angelico (Guido di Pietro)
Museum: The Metropolitan Museum of Art, New York
Inventory number:43.98.5
Date: ca. 1420–23
Technique: Tempera
Support: Wood, gold ground
Dimension: 63.8 x 48.3 cm
Source: Fra Angelico (Guido di Pietro) | The Crucifixion | The Metropolitan Museum of Art

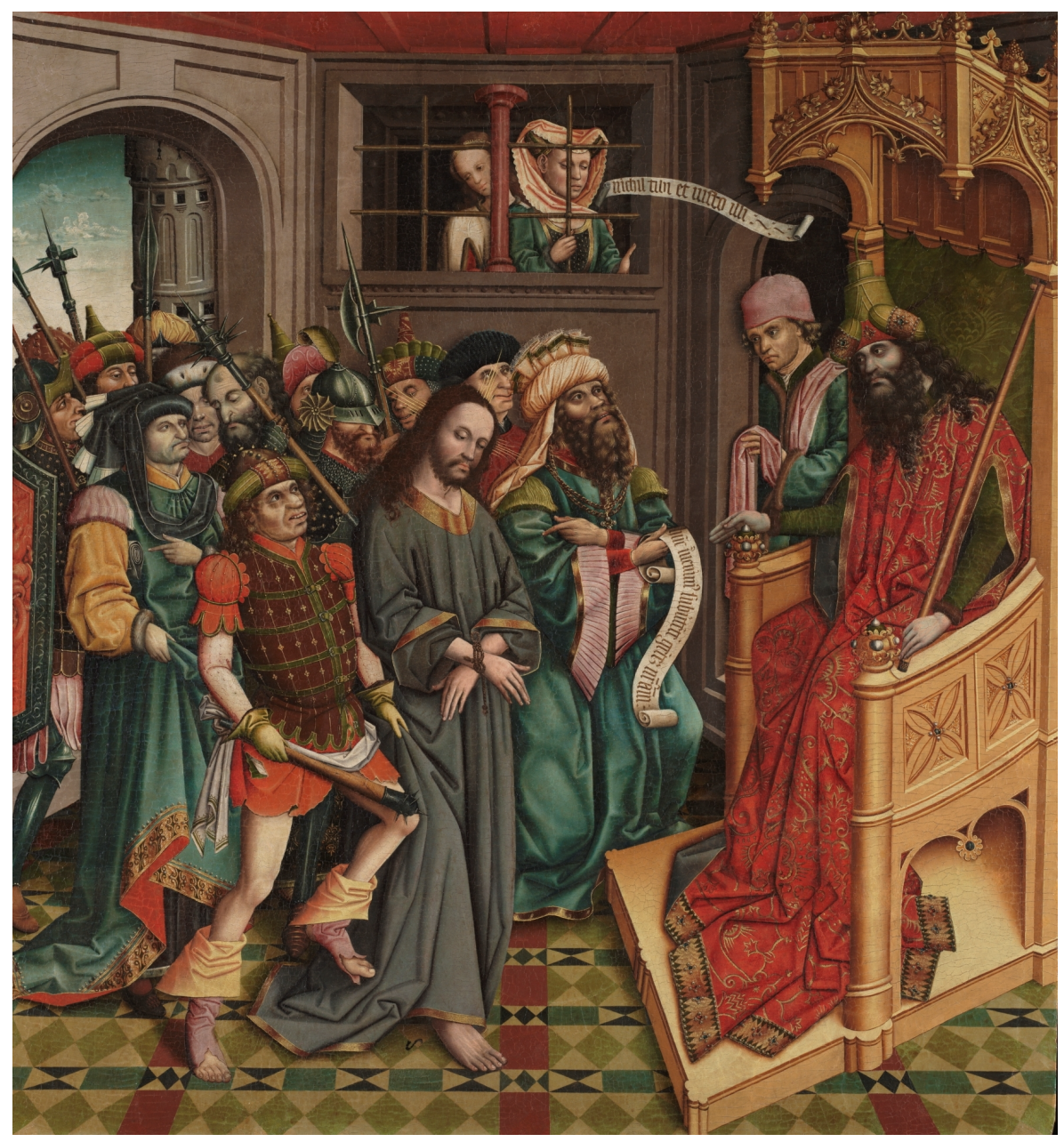

Title: Christ before Pilate
Author: Maestro de Lupiana
Museum: Prado Museum, Madrid
Inventory number: P007157
Date: 1450 - 1460
Technique: Tempera
Support: Panel transferred to canvas
Dimension: 186 cm x 175 cm
Source: https://www.museodelprado.es/en/the-collection/art-work/christ-before-pilate/d28cde19-ad29-4aea-b7b8-153479b8efc2?searchid=7710bd29-c2dd-62a3-e080-35e746a83028
